# Linking the metals to metabolism in recurrent pregnancy loss through untargeted metabolomics and machine learning

**DOI:** 10.3389/fendo.2025.1679190

**Published:** 2025-12-08

**Authors:** Cai Liu, Ling Liu, Bingqing Ran, Yuejuan Wu, Fang Wang

**Affiliations:** 1 Department of Reproductive Medicine, The Second Hospital of Lanzhou University, Lanzhou, Gansu, China; 2 Department of Reproductive Medicine, Gansu University of Chinese Medicine Clinical Medical College of Integrated Chinese and Western Medicine, Lanzhou, Gansu, China

**Keywords:** recurrent pregnancy loss, metals, association, machine learning, metabolism

## Abstract

**Background:**

The association between recurrent pregnancy loss (RPL) and environmental exposure has attracted increasing attention. However, associations between RPL and metal exposure in northwestern China remained unclear.

**Methods:**

This case-control study (318 RPL women, 326 controls) investigated associations between serum metal concentrations and RPL. Five machine learning algorithms identified significant variables. Bayesian kernel machine regression (BKMR) and quartile g-computation (Qgcomp) models assessed the combined effects of metal mixtures on RPL risk. Untargeted metabolomics integrated with metal exposure data explored potential mechanisms underlying metal-induced disruption.

**Results:**

Compared to controls, RPL women exhibited higher BMI (P<0.001) and elevated serum Ti, Cu, and Se levels (P<0.05), while controls had higher Li, V, Cr, Sr, Pb, Ni, Zn, and Fe (P<0.05). Machine learning algorithms (AUC = 0.99-1.0) identified V, Li, Cr, Ti, and Ni as top five discriminative metals. Mixture analyses (BKMR/Qgcomp) revealed a significantly increased RPL risk with mixed metals (β=0.37, 95% CI: 0.31–0.42). Ti contributed positively to this risk, whereas V contributed negatively after adjusted for con-founders. Metabolomic analysis in a subset (n=100) linked these metals primarily to perturbations in purine metabolism, pantothenate and CoA biosynthesis, retinol metabolism, and ubiquinone/terpenoid-quinone biosynthesis.

**Conclusion:**

Our study provides valuable insights into the metabolic and environmental factors associated with RPL.

## 1 Introduction

Pregnancy loss, defined as the spontaneous cessation of pregnancy before fetal viability (from conception to 24 weeks of gestation) (1), represents a significant reproductive health issue. Recurrent pregnancy loss (RPL), clinically defined as two or more losses, affects approximately 5% of women of reproductive age (2). Current epidemiological data are likely an underestimate, as cultural stigma surrounding early pregnancy loss contributes to substantial underreporting. Compounding this concern is clinical evidence indicating that a prior loss independently increases the risk of subsequent miscarriage by 20–40% (3). Beyond the physical implications, individuals affected by RPL frequently experience profound psychological sequelae, including persistent guilt, social withdrawal, and complex grief, which impose a substantial psychosocial burden on families (4). Consequently, the accurate identification of underlying causes and the development of targeted interventions remain critical clinical priorities. Despite advances in reproductive medicine, contemporary diagnostic protocols fail to identify a causative factor in 40–50% of RPL cases (5), highlighting a substantial gap in our understanding of its pathophysiology. This diagnostic ambiguity underscores the urgent need for intensified research to elucidate the molecular mechanisms and develop novel therapeutic strategies.

In light of the unknown etiology in many cases, research has increasingly turned to the role of environmental factors, particularly exposure to heavy metals, as potential contributors to pregnancy loss. A systematic review and meta-analysis have indicated that exposure to certain metals during early pregnancy is correlated with an elevated incidence of spontaneous abortion (6). Concurrently, the integration of metabolomics—the comprehensive analysis of metabolic profiles under various physiological and pathological states—into environmental science has provided a powerful framework for understanding biological responses to exposures (7, 8). This approach offers valuable insights into perturbations within metabolic networks, revealing how organisms respond to environmental stressors.

While recent studies have begun to apply these tools, a specific focus on RPL is lacking. For instance, Shi et al. (9) utilized metabolomics and machine learning to investigate environmental exposures in single early pregnancy loss, yet no study has concentrated specifically on the recurrent form. Our study aims to address this gap. We seek to identify associations between serum metal exposure and RPL and to explore the potential mechanisms by which metals disrupt critical biological pathways. This is now feasible thanks to recent advances in metabolomic profiling techniques, which enable the identification of specific metabolic perturbations induced by environmental exposures.

Furthermore, machine learning (ML) has emerged as a transformative tool in predictive medicine, capable of analyzing large, high-dimensional datasets and uncovering complex patterns beyond the reach of conventional statistics. Modern ML algorithms demonstrate superior performance in integrating multi-modal data, making them ideally suited for deciphering the intricate interplay between environmental exposures and metabolic dysregulation.

To this end, our study employs a combined metal exposure and metabolomic approach to investigate two critical aspects: 1) the specific metals exposure profiles associated with RPL, and 2) the complex interplay between these exposure profiles and dysregulated metabolic pathways during early gestation. This dual-focused investigation aims to provide novel insights into both biomarker discovery and the biological mechanisms underlying metal-associated recurrent pregnancy complications.

## 2 Materials and methods

### 2.1 Study design and participants

This study was conducted at the Second Hospital of Lanzhou University from January, 2021 to December, 2023. The study is an ongoing cohort study, as detailed in a previous publication (10), approved by the institutional review board of the Second Hospital of Lanzhou University (2019A-231) on October 29, 2019. All participants have signed the informed consent and lived in the northwestern part of China. RPL was defined as the occurrence of two or more pregnancy losses before reaching 24 weeks of gestation. Inclusion criteria for RPL were: (1) Age between 18 and 45 years; (2) With two of more pregnancy losses before, including chemical pregnancy; (3)Those with known genetic abnormalities and antiphospholipid syndrome (APS) were excluded. Healthy control was defined as having at least a live birth and no history of pregnancy loss before. The control group comprised 318 women with at least one live birth and no history of pregnancy loss. Control participants were recruited during the same period. Inclusion criteria for controls were: (1) age between 18 and 45 years; and (2) Single live birth. Exclusion criteria for controls were: (1) any history of pregnancy loss; and (2) any of the exclusion criteria for RPL.

### 2.2 Sample collection

All serum samples were collected at baseline enrollment (prior to pregnancy attempts), ensuring preconception heavy metals exposure assessment. The serum was obtained by centrifuging whole blood samples at 4°C and 3000 rpm for 10 minutes within one hour of collection. Subsequently, all serum samples were frozen at −80°C until elemental measurement.

### 2.3 Assessment of serum metals

Blood samples were diluted with nitric acid solution and directly injected for analysis. Inductively Coupled Plasma Mass Spectrometry (ICP-MS) was employed to determine the concentrations of lithium (Li), aluminum(Al), cadmium (Cd), thallium (Tl), lead (Pb), vanadium (V), chromium (Cr), cobalt (Co), nickel (Ni), copper (Cu), zinc (Zn), iron (Fe), arsenic (As), titanium(Ti), strontium (Sr), and selenium (Se). Experimental Procedure: The blood samples were vortexed to ensure homogeneity. A precise volume of 0.25 mL of the blood sample was transferred into a polyethylene tube, followed by the addition of 4.75 mL of nitric acid-Triton X-100 solution. The mixture was thoroughly homogenized and prepared for analysis. Concurrently, deionized water (0.25 mL) was used in place of the blood sample and processed identically to serve as a reagent blank. If the metal content in the blood exceeds the detection range of this method, the sample can be further diluted and analyzed.

#### 2.3.1 Instrument conditions

Plasma power: 1.35 kW;Peristaltic pump speed: 15 r/min;Cool gas flow rate: 10.5 L/min;Auxiliary gas flow rate: 1.35 L/min;Nebulizer gas flow rate: 0.94 L/min. Other parameters were adjusted to their optimal settings according to the instrument’s manual. Quality Control during Detection: During the experimental detection process, a parallel sample analysis was performed for every 10 samples, with a requirement that the relative deviation between parallel samples be less than 20%. For every 100 samples, 1–2 spike recovery experiments were conducted, with a requirement that the recovery rate falls within the range of 80% to 120%.

The detection limit (LOD) values for each heavy metal are described in **Supplementary Table S1**. The concentration of blood heavy metals below the lower LOD was replaced with the LOD divided by √2. In the present study, the detection rates of 12 heavy metals were all >50%, those with <50% detection rates (Cd, Tl, Co, and As) were excluded in this article.

### 2.4 Untargeted metabolomics profiling

UHPLC–MS/MS analysis was performed using a Vanquish UHPLC system coupled with an Orbitrap Q Exactive™ HF-X mass spectrometer (Thermo Fisher, Germany) at Novogene Co., Ltd. The specific method was reported in a previous study (11). Separation was achieved using a Hypersil Gold column (100 × 2.1 mm, 1.9 μm) with a 12-minute linear gradient at a flow rate of 0.2 mL/min. The mobile phase for positive ionization mode consisted of water with 0.1% formic acid (eluent A) and methanol (eluent B), while for negative mode, 5 mM ammonium acetate (pH 9.0, eluent A) and methanol (eluent B) were used. The gradient program was as follows: 2% B (1.5 min), increase to 85% B (3 min), then to 100% B (10 min), return to 2% B (0.1 min), and hold at 2% B (12 min). MS detection was performed in both positive and negative modes with the following settings: spray voltage 3.5 kV, capillary temperature 320 °C, sheath gas 35 psi, auxiliary gas 10 L/min, auxiliary gas heater 350 °C, and S-lens RF level 60. High-energy collision dissociation (HCD) was applied across an m/z range of 100–1500 using three collision energies (20, 40, and 60 V). Quality control (QC) samples were analyzed alongside study samples to ensure data reliability through correlation assessment of QC injections. All samples were processed in a single analytical batch.

### 2.5 Covariates

Based on the patients and healthy controls in this study, the following covariates were selected: age, body mass index (BMI), waist-to-hip ratio (WHR), education level (≤ high school and > high school) and ethnicity (Chinese Han and the minority).

### 2.6 Statistical analysis

Group differences between the two groups were assessed using statistical tests appropriate for the data type, including the Student t-test, Mann-Whitney U test, or chi-square test. Continuous variables were described by mean value ± standard deviation (SD), while categorical variables were expressed as n (%). For subsequent analyses, metals concentrations were subjected to a natural-log transformed to improve their distribution normalization. Spearman’s rank correlation analysis was applied to illustrate the pairwise correlations between metals. Spearman’s correlation coefficients were classified as strong (r > 0.8), medium (≧ 0.3 and ≤ 0.8) and weak (r < 0.3) (12).

Subsequently, Light Gradient Boosting Machine(Light GBM), K-Nearest Neighbors(KNN), Support Vector Machine(SVM), Random Forest (RF), and Extreme Gradient Boosting (XGBoost) models were used to screen out more significant metals contributing to RPL.

In addition, quantile-based g computation (Qgcomp) was employed to investigate the combined impact of mixtures of metals. This approach yielded unbiased outcomes with appropriate confidence intervals for actual sample sizes, enabling the included elements to be positively or negatively associated with the results (13). Furthermore, the Bayesian Kernel Machine Regression (BKMR) model was employed, BKMR is a sophisticated statistical approach designed to assess the health impacts of multi-pollutant mixtures. It offers a flexible framework that can accommodate non-linear relationships and interactions among components of the mixture (14). The present study aims to examine the non-linear relationship between exposure and outcome by utilizing a single variable and the exposure-response cross-section, while maintaining other variables at their median values. The bivariate exposure-response curve illustrates how the interaction of various components can be interpreted as the potential interaction between one chemical substance, modified by another chemical substance for the 25th, 50th, and 75th percentiles (with all other variables fixed at their medians). The association plot of combined effects with outcomes demonstrates the variability in estimated results when all exposure variables are simultaneously set at different percentiles compared to when they are fixed at their medians. The combined effects of metal mixtures on recurrent pregnancy loss were investigated. The estimate of the BKMR model was calculated after 1,000 iterations using R package (“bkmr”).

In our study population, missing values for baseline characteristics variables were <10% and handled using Multiple interpolation in the models. Statistical analyses were conducted using IBM SPSS version 27.0. P values of less than 0.05 were considered statistically significant.

## 3 Results

### 3.1 Baseline characteristics of study participants and heavy metals distribution

The baseline characteristics of the study participants were presented in **Table 1**. The study comprised 318 RPL cases and 326 healthy controls. As showed in the **Table 1**, the age was higher in the control group (30.72 vs. 33.53 years), whereas BMI was comparable between the two groups (22.65 vs. 22.18 kg/m^2^, *P*>0.05). In addition, education level in the control group was higher (*P* < 0.001), whereas no significant difference was observed in the ethnicity between RPL and controls. Regarding metal concentrations, Li, V, Cr, Sr, Pb, Ni, Zn, and Fe were significantly higher in the control group (*P* < 0.05). In contrast, Ti, Cu and Se concentration were elevated in the RPL group (*P* < 0.05). However, no significant difference in Al concentration was observed. The quartiles for each metal were presented in **Supplementary Table S1**.

**Table 1:** Baseline characteristics of study participants.

Variable	Recurrent pregnancy loss	Control	*P*-value (t/u test)
n	318	326	
Age	30.72 ± 3.85	33.53 + 3.73	**<0.001**
BMI(kg/m^2^)	22.65 ± 3.07	22.18 ± 3.31	0.074
PLs	2.57 ± 0.91	–	–
WHR	0.86 ± 0.06	0.83 ± 0.06	**<0.001**
Education			**<0.001**
≦High school	94(29.6)	29(8.9)	
>High school	182(57.2)	296(90.8)	
Unknown	42(13.2)	1(0.3)	
Ethnicity			**0.014**
Chinese Han	255(80.2)	315(97.7)	
Minority	22(6.9)	11(2.3)	
Unknown	41(12.9)	0(0.0)	
Li(ug/L)	1.03 ± 0.70	2.19 ± 0.51	**<0.001**
Al(ug/L)	6.11 ± 0.81	6.18 ± 0.89	0.205
Ti(ug/L)	4.98 ± 0.22	4.62 ± 0.32	**<0.001**
Sr(ug/L)	4.41 ± 0.45	4.51 ± 0.44	**0.008**
V(ug/L)	0.84 ± 0.31	1.30 ± 0.19	**<0.001**
Cr(ug/L)	3.17 ± 0.43	3.88 ± 0.47	**<0.001**
Pb(ug/L)	1.65 ± 0.58	1.96 ± 0.73	**<0.001**
Ni(ug/L)	1.59 ± 1.20	2.47 ± 0.93	**<0.001**
Cu(ug/L)	7.02 ± 0.35	6.88 ± 0.28	**<0.001**
Zn(ug/L)	6.87 ± 0.46	6.92 ± 0.34	**0.029**
Fe(ug/L)	7.28 ± 0.36	7.42 ± 0.41	**<0.001**
Se(ug/L)	4.75 ± 0.29	4.69 ± 0.36	**0.010**

Statistically significant values (p < 0.05) are highlighted in bold.

Metal concentrations were presented as Ln-transformed values.

### 3.2 Correlations among exposure to multiple metals

**Figure 1:**
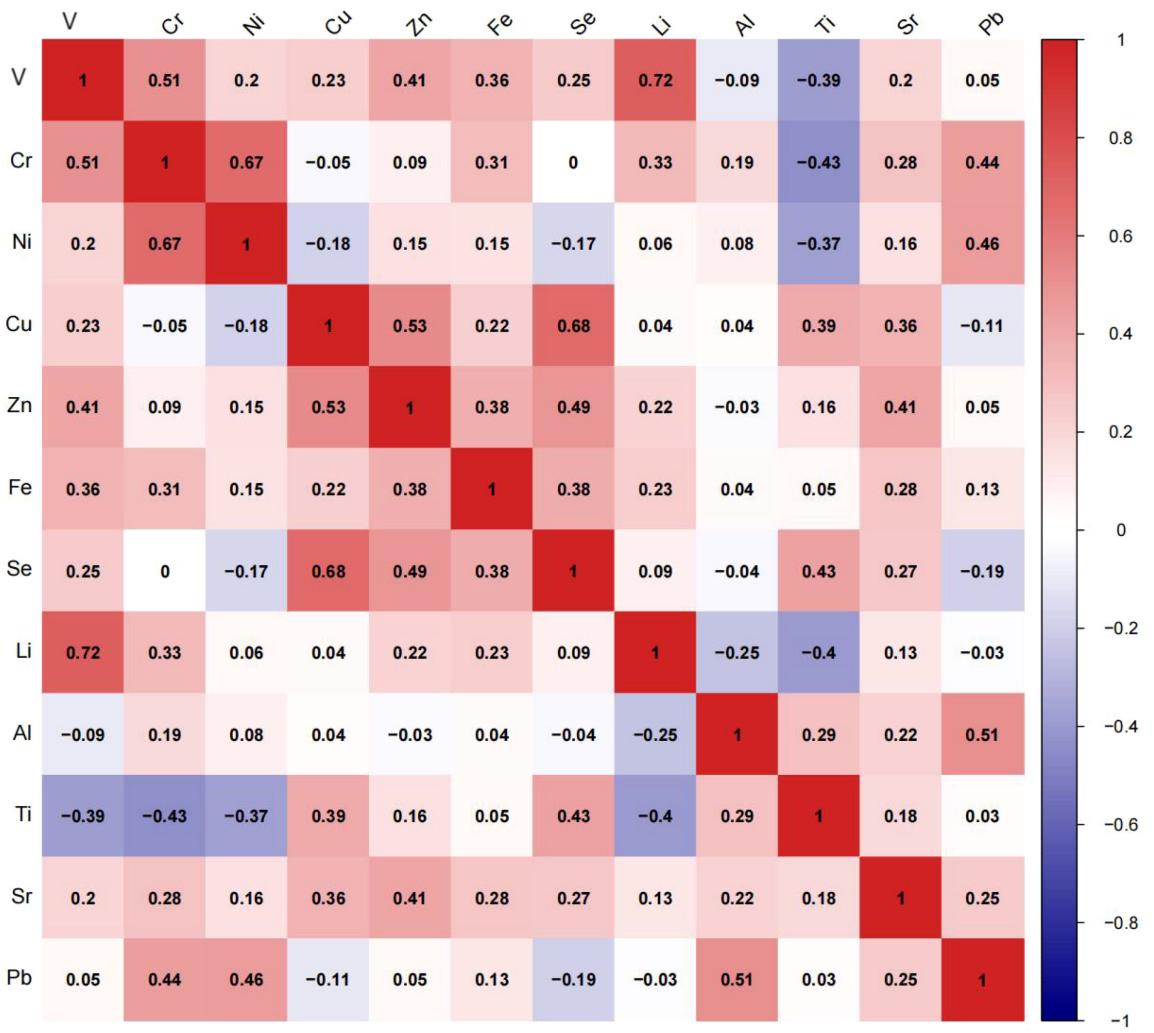
Heatmap of Spearman correlation analysis in all participants.

Most of the metals showed significant but weak correlations as presented in **Figure 1**, the correlation between these metals after ln-transformed was showed by Spearman’s correlation coefficients. There was a moderate correlation between Li and V (r = 0.72), Li and Ti (r = -0.40), Li and Cr (r = 0.33), Ti and V (r = - 0.39), Ti and Cr (r = - 0.43), Ti and Ni (r = - 0.37), Ti and Cu (r = 0.39), Ti and Se (r = 0.43), Sr and Cu (r = 0.36), Sr and Zn (r = 0.41), Sr and Fe (r = 0.28), Pb and Cr (r = 0.44), Pb and Ni (r = 0.46), V and Cr (r=0.51), V and Zn (r = 0.41), Cr and Ni (r = 0.67), Cr and Fe (r = 0.31), Cu and Zn (r = 0.53), Cu and Se (r = 0.68), Zn and Fe (r = 0.38), Zn and Se (r = 0.49), Fe and Se (r = 0.38).The correlations between others were relatively weak.

The colors present the correlation, with red being positive and blue being negative.

### 3.3 Variable selection on ML algorithms

**Figure 2:**
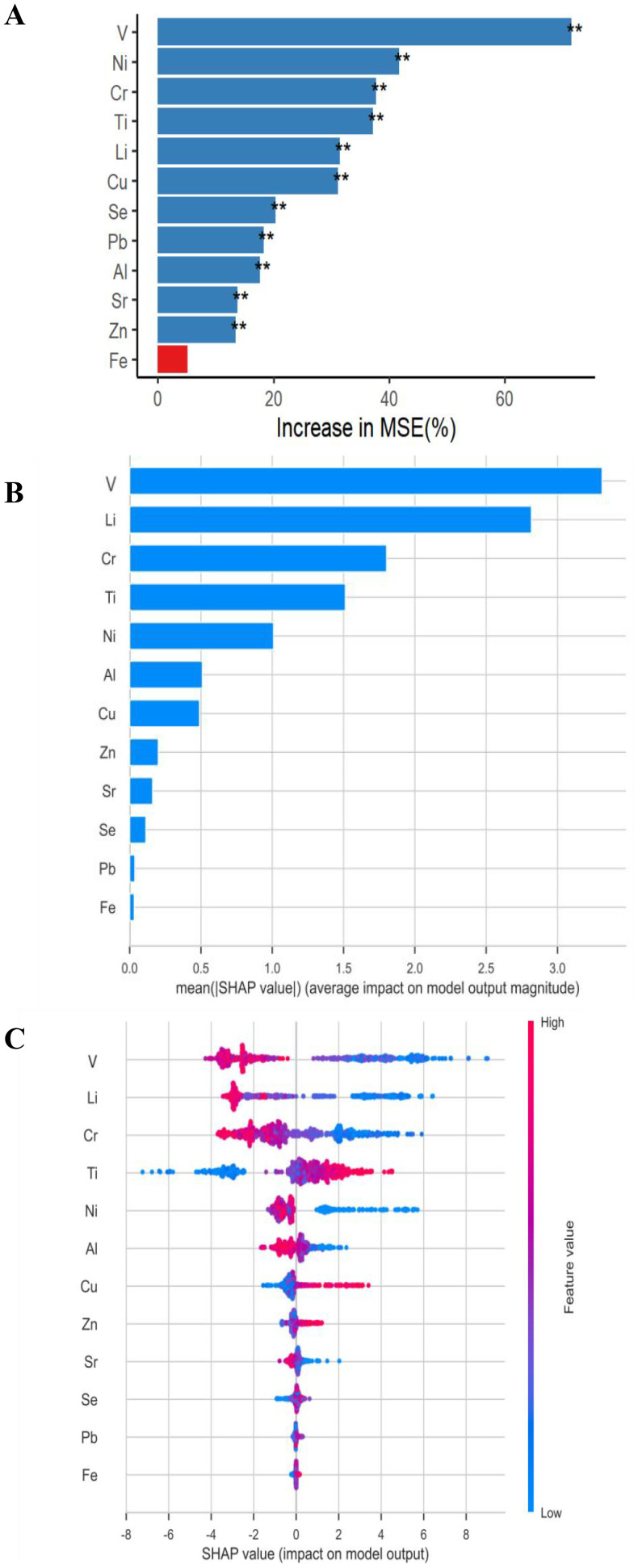
Variables importance rankings measuring the association between metals exposure and RPL risk based on RF and LightGBM models interpreted with the SHAP approach. **(A)** RF variable importance. **(B)** SHAP summary bar plot. **(C)** SHAP summary dot plot. The potential of RPL risk increases with a feature’s SHAP value. Each patient’s SHAP value is represented by a dot, and the colors of the dots represent the actual values of the features for each patient, with red representing a higher feature value and blue representing a lower feature value. The dots are stacked vertically to demonstrate density. ** for p < 0.01.

The dataset was split into training and testing sets with an 70% and 30% ratio, respectively. Five ML algorithms were used to screen out variables. Light GBM, KNN, SVM, RF, and XGBoost were constructed to predict the risk of RPL. During training, we used 5-fold cross-validation to ensure robustness. As presented in **Supplementary Figure S1**, Light GBM and XGBoost performed well, with ROC results showing an AUC of 1.0, along with RF, KNN and SVM (AUC = 0.99) (**Supplementary Figure S1**). The variable importance of the RF and LightGBM models were illustrated in **Figure 2**. The LightGBM model identified five more important variables, ranked in descending order of importance as follows: V, Li, Cr, Ti, and Ni. The best model for predicting RPL was identified by comparing performance metrics on the test set. To interpret the final model, the SHAP method was employed to calculate the contribution of individual exposures to the predictions. The SHAP summary plots (**Figures 2B, C**) provide a global interpretation of the model at the feature level and a local interpretation at the individual level, respectively. The contribution of each feature to the model is assessed using average SHAP values, presented in descending order. The top five variables based on average SHAP values were V, Li, Cr, Ti, and Ni, which corresponded to the variables identified by the RF model. Furthermore, the SHAP dependency plot (**Figure 2C**) illustrated how each individual feature influences the model’s output. Higher feature values were associated with a greater likelihood of RPL, with red and blue dots representing higher and lower values, respectively. These SHAP dependency plots visually demonstrated the impact of individual features on the model predictions.

### 3.4 Mixed exposure risk for RPL

#### 3.4.1 BKMR model to evaluate the associations of metal mixtures and RPL

**Figure 3:**
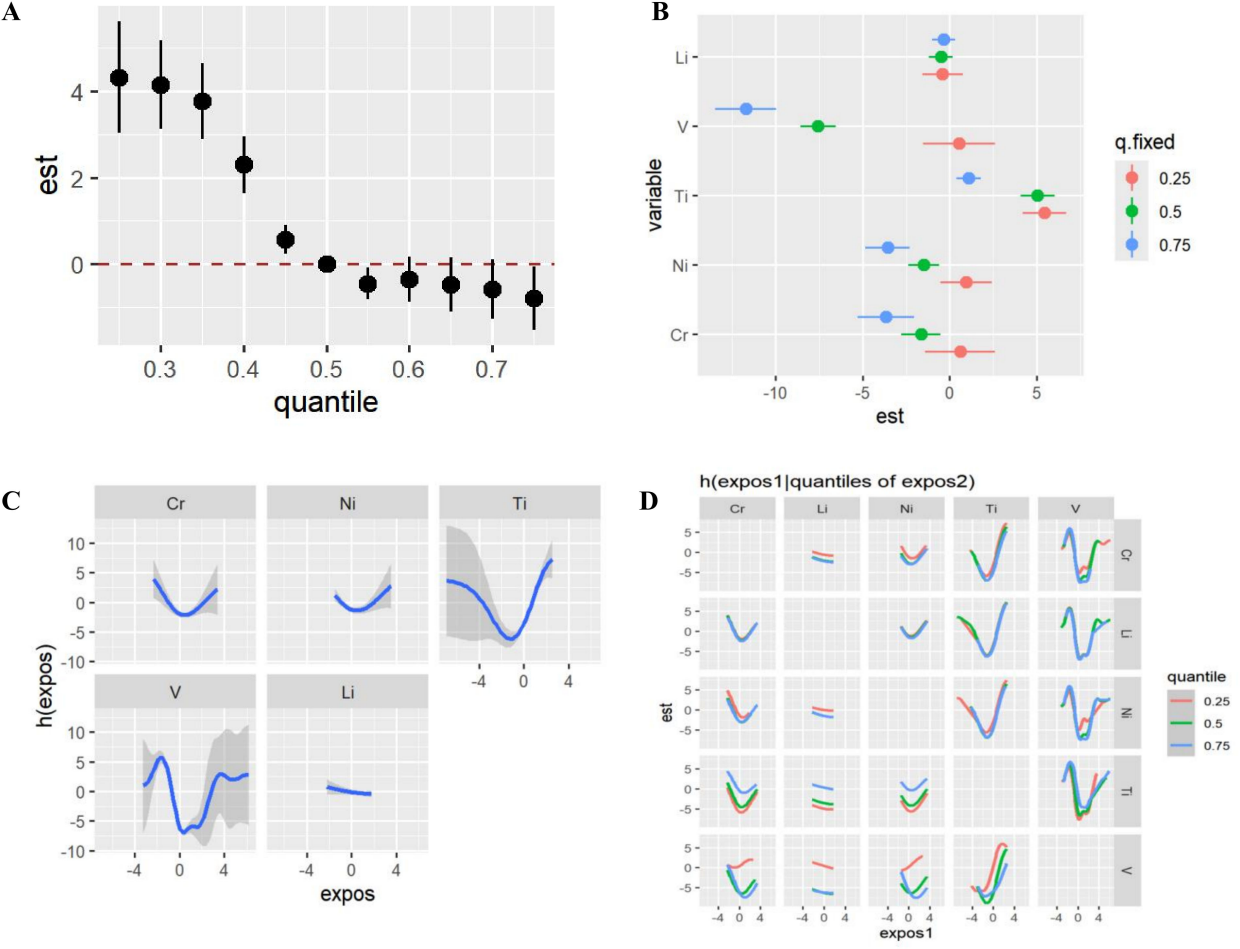
Analysis using the BKMR method examines the combined impact of exposure on RPL risk, where h(Z) reflects the relationship between metals and a hidden continuous marker indicating RPL status. **(A)** the total effect of the heavy metals on RPL, comparing metals at certain percentiles with those at the 50th percentile. **(B)** summarizes the univariate risk for each chemical by quartiles. **(C)** Exposure-response function between 5 heavy metals and RPL estimated by BKMR models. Models were adjusted for age, BMI, WHR, education and ethnicity. **(D)** the graph compares the dose response curves for each heavy metal with the risk of RPL, when all other metals were at the 25th (red), 50th (green), and 75th (blue) percentiles. Models were adjusted for age, BMI, WHR, education level and ethnicity.

The joint effect of metals on RPL was displayed in **Figure 3A**. The risk of RPL exhibited an increased risk with the decrease of metal concentration compared to the 50th percentile. The combined effect of exposure values was statistically significant. **Figure 3B** showed that the positive impact of Ti on RPL increases as the percentile of the other four metals decreases. In addition, the negative effect of V on RPL increased with the increase of the percentile of other metals. Similarly, the negative effect of Cr on RPL increased with the increase of the percentile of other heavy metals. **Figure 3C** showed the exposure-response function trend of 5 metals, indicating the association between metals and potential continuous markers of RPL risk while maintaining all other metals at median levels. Ti, V, Cr and Ni showed an asymmetric U-shaped association. Ti had a significant positive effect, and its association with RPL was stronger at lower levels of other metals. The negative association between V and RPL risk was significant when other metals were fixed at the 75th percentile. The double exposure variable curve reveals a significant interaction between V, Ti and the other four metals, and none of the remaining interactions was significant (**Figure 3D**).

#### 3.4.2 Qgcomp model to evaluate the associations of metal mixtures and RPL

**Figure 4:**
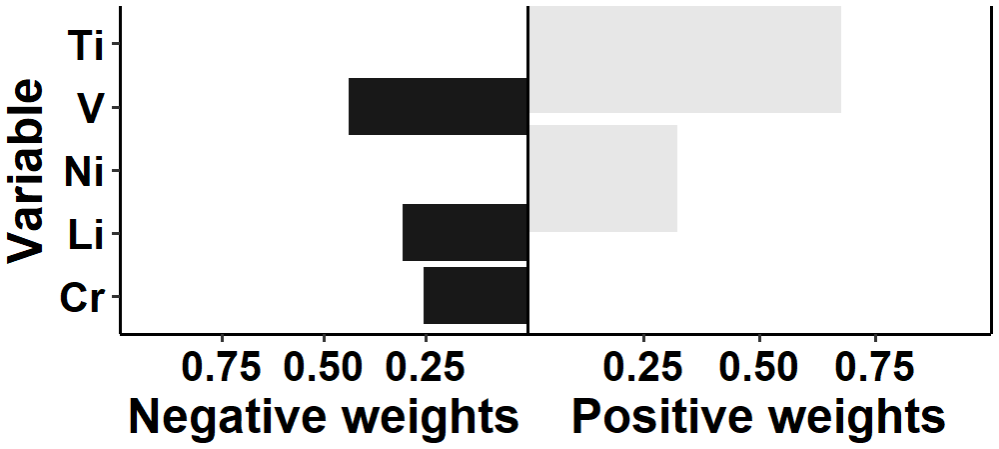
Weighting of the effect of individual metal on RPL.

Qgcomp analyses exhibited the estimated exposure weights in the positive and negative directions, respectively after adjusted for age, BMI, WHR, education, ethnicity, and other seven elements (Fe, Sr, Zn, Cu, Se, Pb, Al). The joint weighted value of serum exposure was significantly associated with RPL status in total [0.37 (0.31, 0.42)]. **Figure 4** illustrates the scale weights calculated by qgcomp for each heavy metals. Notably, among the RPL, the weights for Ti and Ni were positive. On the other hand, the weights for Li, V and Cr were negative.

### 3.5 Baseline characteristics of participants for the combined analysis of heavy metals exposure and untargeted metabolism

Among 318 RPL women, 50 had conducted serum untargeted metabolic profiling. Therefore, we included 100 participants in the integrated analysis for both metals exposure and untargeted metabolic profiling: 50 women who had experienced RPL and 50 age-matched healthy controls (1:1 ratio). As presented in **Supplementary Table S2**, compared with control group, the pregnancy loss group had a similar mean age (30.38 vs. 31.12, *P* = 0.241) and BMI (22.86 kg/m² vs. 22.29 kg/m², *P* = 0.424). Furthermore, no significant difference was observed in blood pressure (SBP: 114.50 mmHg vs.110.10 mmHg, *P* = 0.072; DBP: 72.44 mmHg vs. 71.18 mmHg, *P* = 0.507). However, education level was higher in control group (> High school: 66.0% vs. 94.0%, *P* < 0.001). Besides, WHR was higher in the pregnancy loss group (0.86 vs. 0.82, *P* < 0.001). No significant difference was found in the distribution of ethnicity (*P =* 0.242).

### 3.6 OPLS-DA model and permutation test

**Figure 5:**
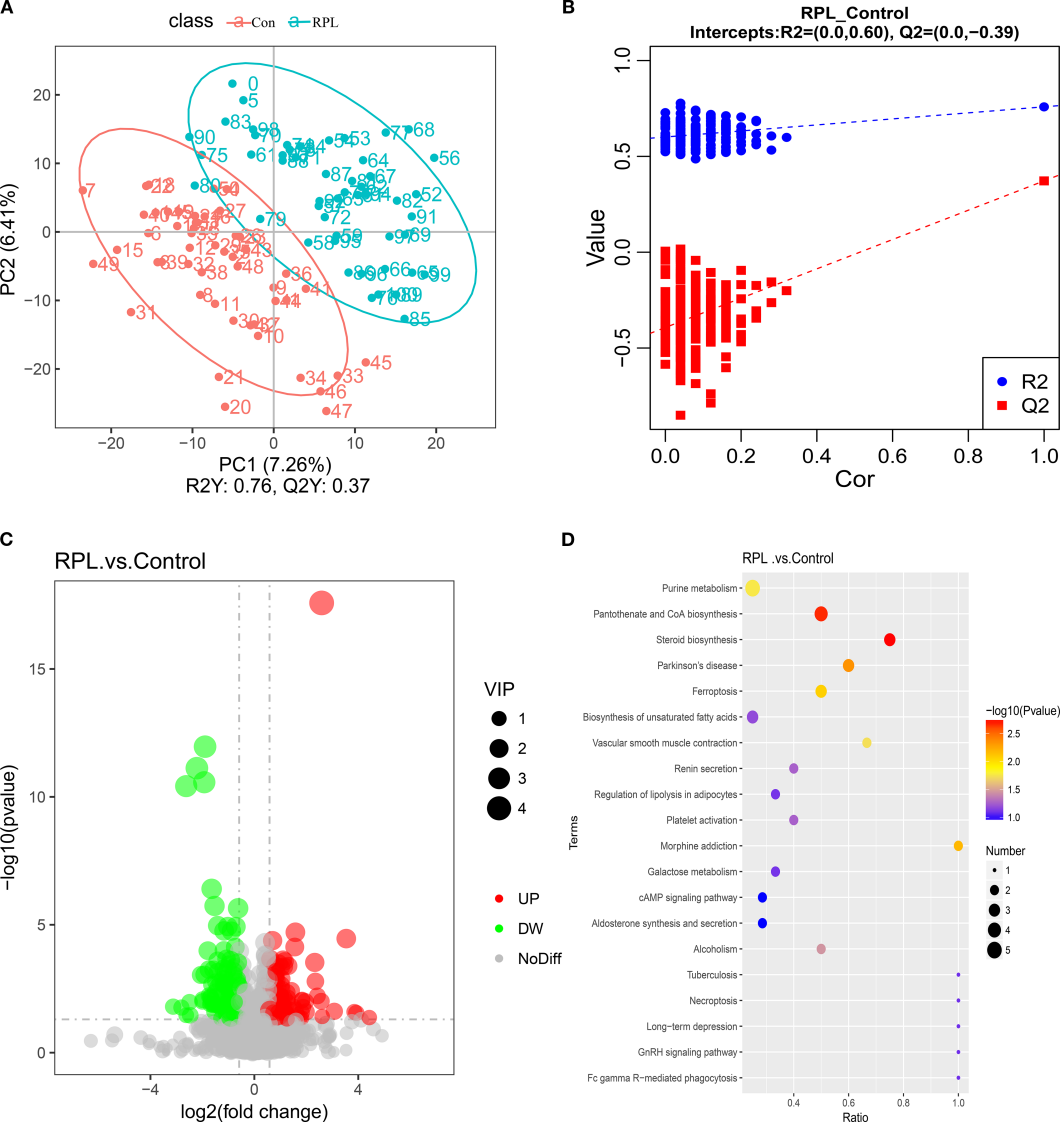
Metabolic analysis. **(A)** OPLS-DA score plot showing the separation between the PL group and the control group based on their metabolic profiles. **(B)** Permutation test results with 200 iterations to evaluate the robustness of the OPLS-DA model. **(C)** Volcano plot of metabolites. **(D)** Pathway enrichment analysis bubble plot.

Metabolomic profiling was conducted on the Novogene platform (https://magic.novogene.com). A total of 2473 metabolites in serum were annotated using both positive and negative ionization modes. The pregnancy loss group showed significant up-regulation (116 metabolites) and down-regulation (120 metabolites) compared to the healthy controls (VIP >1, *P* < 0.05) (**Figure 5A**). Quality control samples clustered closely in the PCA score plots, demonstrating the method’s stability. However, the samples did not exhibit clear separation. To further explore the relationship between metabolite profiles and sample groups, OPLS-DA was subsequently employed for supervised modeling and classification. **Figure 5A** illustrates the OPLS-DA model. The OPLS-DA plot revealed a separation between the control and RPL group, indicating distinct metabolic profiles. The x-axis (PC1) explained 7.26% of the variance between the two groups, whereas the y-axis (PC2) explained 6.41% of the variance within each group (**Figure 5B**). The OPLS-DA score plot showed that the control group and the RPL group are separated significantly in the model space, suggesting that there are certain differences between the two groups. The R^2^Y value of the model is 0.76, indicating that the model can fit the grouping information well. The Q^2^Y value is 0.37, and the predictive ability is average. The permutation test results of the OPLS-DA model showed that the Q^2^ and R^2^ values of the original model were significantly higher than those of the model generated by random permutation, and the cut-off of Q^2^ is -0.39 (**Figure 5B**), which is lower than 0. This indicates that the model has not undergone overfitting and has good stability and reliability. Therefore, the inter-group differences revealed by the model are reliable and can be used for subsequent biological analysis and biomarker screening.

Among the significantly altered metabolites (**Figure 5C**), metabolic pathways were conducted using the KEGG database. These pathways are visualized in the bubble plot (**Figure 5D**), where larger and more intensely colored bubbles represent pathways with higher impact and significance. Pathway impact analysis highlighted several significantly affected pathways, such as purine metabolism, pantothenate and CoA biosynthesis, steroid biosynthesis, Parkinson’s disease, and ferroptosis.

### 3.7 Correlation between metals and untargeted metabolism

Firstly, we investigated the associations between variables and metals, as well as variables and the selected five metabolites.As showed in **Supplementary Tables S4**, **S5**, both metal concentrations and metabolomic results showed no or weak relationship with covariates (age, BMI, WHR, education level and ethnicity). To further explore the associations between metals and untargeted metabolism, we conducted Spearman correlations between these five more significant metals and 236 different metabolites. As presented in **S Supplementary Tables S6**-**S10**, significant associations were identified between 169 metabolites and V(*P* < 0.05), 153 metabolites and Li (*P* < 0.05), 57 metabolites and Ti (*P* < 0.05), 53 metabolites and Cr(*P* < 0.05), and 11 with Ni (*P* < 0.05). Additionally, KEGG analysis (https://dev.metaboanalyst.ca/MetaboAnalyst/) with these significant correlated metabolites was conducted to identify the specific metabolic pathway with five metals. As presented in **Supplementary Figures S2A–E**, the top two metabolic pathways significantly associated with each metal were identified as follows, V: purine metabolism, pantothenate and CoA biosynthesis. Ti: retinol metabolism, ubiquinone and terpenoid-quinone biosynthesis. Li: purine metabolism, galactose metabolism. Cr: retinol metabolism, arginine/proline metabolism. Ni: biosynthesis of unsaturated fatty acids, steroid hormone biosynthesis. Among these, purine metabolism and retinol metabolism were closely correlated with two metals.

To further investigate these associations, we performed linear regression analyses between the five metals and their respective top five significantly correlated metabolites (identified by Spearman analysis, *P* < 0.05).

As presented in **Supplementary Figure S3**. V exhibited a negative correlation with panthenol, while positive correlations were observed between V and 7-alpha-carboxy-17-alpha-carboxyethylandrostan lactone phenyl ester, Tretinoin, (+/-)-Cannabichromeorcin, and (+/-)-8-HEPE. In contrast, Ti showed a positive correlation with panthenol, but negative correlations were observed between Ti and 7-alpha-carboxy-17-alpha-carboxyethylandrostan lactone phenyl ester, Tretinoin, (+/-)-Cannabichromeorcin, and (+/-)-8-HEPE. Li demonstrated positive relationships with Tretinoin, (+/-)-Cannabichromeorcin, and (+/-)-8-HEPE, while negative correlations were found between Li and dipropylene glycol dimethyl ether as well as Panthenol. Additionally, Cr was negatively associated with both panthenol and guanosine 3’,5’-cyclic monophosphate, but positively correlated with N-tetradecanamide, 2,4-dimethylbenzaldehyde, and homotaurine. Ni showed a negative correlation with desoxycortone and a positive association with acetylcysteine, 2-aminobenzenesulfonamide, N-{5-[(dimethylamino)sulfonyl]-2-methylphenyl}cyclohexanecarboxamide, and obscurolide A1.

## 4 Discussion

This study introduces a machine learning-based data analysis framework to investigate the association between metals exposure and RPL risk by integrating metabolomic data. Our findings revealed an overall adverse effect of mixed-metal exposure on RPL risk, with Ti as the primary positive contributor and V identified as the primary negative contributor. Significantly, this is the first study to integrate serum metal profiling with metabolomic analysis. It demonstrates that metal exposure dysregulates key metabolic pathways, namely purine metabolism, pantothenate and CoA biosynthesis, retinol metabolism and ubiquinone/terpenoid-quinone biosynthesis, which are implicated in the pathogenesis of RPL.

### 4.1 Association between heavy metals exposure and RPL risk

In the variable selection using ML models, V, Ti, Li, Cr and Ni are considered to be the key exposures that differentiate RPL cases from healthy controls. Results from the BKMR and Qgcomp models consistently support a negative association of mixed metals exposure with RPL risk. Zhang et al. (15) reported that calcium and selenium exposure were consistently negatively associated with miscarriage, while lead exposure was positively associated with miscarriage. However, mixed exposure to multiple metals has not been evaluated. The levels of Cr were lower than those observed in our study, with median levels of 1.74 μg/L for Cr, compared to our study’s levels of 22.28 μg/L in RPL women. However, the levels of V and Ni were higher than those observed in our group, with the median levels of 11.63μg/L for Ni and 0.23 mg/L for V, compared to our study’s levels of 5.34 μg/L for Ni and 2.22 μg/L for V in RPL women. However, there is a paucity of evidence regarding the association between mixed-metals exposure and RPL risk. Furthermore, due to the different environmental exposure and the variations in the specific metals being assessed, as well as disparities in exposure levels, may contribute to the observed differences in results.

This study identified that V as the major contributor to the negative associations with RPL. Vanadium is recognized as an essential trace element, with (16) demonstrating its significance for infant growth, particularly during early pregnancy. This supports our findings. Furthermore, Ti was identified as the major contributor for the positive associations with RPL risk, this was consistent with Roberto et al’s findings, they found that elevated titanium levels were associated with reduced implantation rates and adverse pregnancy outcomes during *in vitro* fertilization (IVF) cycles (17). Several studies have reported associations between titanium exposure and ovarian dysfunction, with titanium dioxide nanoparticles inducing ovarian alterations that reduce embryo development rates and fertility (18, 19). Prenatal exposure to titanium may lead to the accumulation of reactive oxygen species, DNA damage, and activation of signaling pathways such as MAPK, potentially impairing neurotransmission (20). Previous study (21) demonstrated that exposure of human placental explants to titanium dioxide at physiologically relevant concentrations resulted in dysregulation of inflammatory and placental factors. Given that titanium can cross the placental barrier (22), it is crucial to monitor and manage titanium exposure in women experiencing RPL. Cr, as another negative contributor to RPL, is an another essential trace element that plays a critical role in glucose and lipid metabolism. Zhang et al. (23) identified that lower chromium levels were associated with increased offspring body weight and triglyceride levels, while maternal chromium status significantly impacted lipid metabolism in offspring. Yao et al. (24) suggested that chromium supplementation, particularly in conjunction with zinc and selenium, could have beneficial transgenerational effects on glucose homeostasis in female offspring of gestational diabetes mellitus (GDM).

### 4.2 Metabolic dysregulation related to metals exposure and RPL risk

In this study, we identified 236 differentially metabolites between RPL and healthy controls. Among these, purine metabolism and pantothenate and CoA biosynthesis were strongly associated with V, the primary contributor identified in this study. Disturbances in purine metabolism, potentially triggered by metals disruption, compromise DNA synthesis and repair, impairing cellular survival (25). The observed upregulation of guanosine, inosine, adenosine 5’-diphosphate (ADP), and adenosine in RPL women indicates a metabolic shift towards purine salvage pathways. This adaptation likely enables cells to conserve nitrogenous bases under metals-induced stress, highlighting the critical role of purine metabolism in maintaining cellular function under adverse conditions (26). Furthermore, pantothenate and panthenol, as the metabolites of pantothenate and CoA biosynthesis, were upregulated in RPL women. Pantothenate (vitamin B_5_;) exerts its primary functions via incorporation into coenzyme A (CoA) and acyl-CoA thioesters, which are essential for glucose, amino acid, and fatty acid metabolism (27). Mechanistically, pantothenate regulates cellular CoA biosynthesis and protects endothelial function (28). Consequently, pantothenate and its derivatives act as vascular protectors that boost ATP production while reducing malondialdehyde (MDA) — a key lipid peroxidation biomarker (29), underscoring the critical role of pantothenate and CoA biosynthesis in maintaining cellular functions under adverse conditions. Additionally, retinol metabolism and ubiquinone and terpenoid-quinone biosynthesis were closely correlated with Ti, a major positive contributor identified in this study. Retinol metabolism, crucial for immune response, is influenced by oxidative stress, linking it to pathways involved in detoxification and cellular defense (30). The ubiquinone and terpenoidquinone biosynthesis pathway was also significantly associated with Ti. Current research reveals that changes in these pathways impact cellular energy production in bacteria (31). Collectively, these pathways are essential for cellular energy production, hormone synthesis, and oxidative stress regulation – processes critical for maintaining a healthy pregnancy (32, 33). Significantly, the disruption of these key metabolic pathways (purine, pantothenate/CoA, retinol, ubiquinone) in the RPL group suggests a potential mechanistic link between metabolic dysregulation and the effects of metals disruption. These findings highlight a network of interconnected pathways working in concert to restore cellular homeostasis in response to metals-induced stress.

The identification of specific metabolites, such as panthenol, (+/-)-cannabichromeorcin, (+/-)8-HEPE, tretinoin, and 7-alpha-carboxy-17-alpha-carboxyethylandrostan lactone phenylester, as major significant associated metabolites with metals. These metabolites may serve as targets for therapeutic interventions or risk assessment tools in clinical settings. Panthenol, an analogue of pantothenic acid (vitamin B_5_), used in pharmaceuticals, medical devices, and cosmetics (34), is a key precursor in the synthesis of fatty acids that maintain epithelial function (35), showed a significant positive correlation with titanium (Ti) (r = 0.522), indicating that Ti might lead to lipid metabolism disruption. Additionally, Ti was negatively correlated with tretinoin (r= -0.436) and (+/-)8-HEPE(r = - 0.429) (**Supplementary Table S5**). However, V and Li was positively associated with (+/-)8-HEPE(r = 0.595) (r = 0.486) and tretinoin (r = 0.537) (r = 0.436), respectively. Maki et al. (36) reported that 8-HEPE significantly increased the plasma HDL-cholesterol level and decreased the plasma LDL-cholesterol and hepatic triglyceride levels in mice, indicating that (+/-)8-HEPE might improve lipid metabolism (37). Consequently, Ti’s negative correlation with (±)8-HEPE may exacerbate metabolic dysfunction. Tretinoin is a vitamin A derivative widely used in skin treatment. Studies reported that retinoic acidis is integral to the establishment and maintenance of normal pregnancy (38, 39). Metals disruption might result in abnormal production of tretinoin, thus leading to pregnancy loss. Gou et al. (40) reported that 7-alpha-carboxy-17-alpha-carboxyethylandrostan lactone phenylester was higher in developed sepsis compared with those did not develop sepses, suggesting involvement in inflammatory pathology. However, (+/-)-Cannabichromeorcin and 7-alpha-carboxy-17-alpha-carboxyethylandrostan lactone phenylester have not been confirmed the classification. Finally, our results illuminate potential targets for therapeutic intervention. The fact that metal-induced disruption converges on critical pathways for cellular energy, redox balance, and inflammation suggests that supporting these pathways could be a viable strategy. For instance, the upregulation of pantothenate (Vitamin B5) salvage pathways indicates a potential metabolic vulnerability that might be addressed with nutritional support. Similarly, the negative association of Ti with tretinoin and (±)8-HEPE, both implicated in healthy pregnancy maintenance and lipid metabolism, highlights pathways that could be therapeutically modulated to counteract the adverse effects of metal exposure.

### 4.3 Clinical implications

The findings of this study carry significant clinical implications for the risk assessment, prevention, and management of RPL. First, the identification of specific metals as key contributors to RPL risk provides a tangible target for preventive counseling and environmental intervention. Our results pinpoint Titanium as the primary risk metal and Vanadium and Chromium as potential protective elements. This suggests that clinicians could incorporate questions about potential sources of titanium exposure (e.g., certain cosmetics, pharmaceuticals, or environmental pollutants) into routine assessments for women with RPL. Concurrently, ensuring adequate levels of essential trace elements like vanadium and chromium might be beneficial, although their therapeutic supplementation requires further investigation to define safe and effective dosages. Second, the dysregulated metabolic pathways and specific metabolites identified offer a novel foundation for developing diagnostic and prognostic biomarkers. The disturbances in purine metabolism, pantothenate and CoA biosynthesis, retinol metabolism, and ubiquinone biosynthesis provide a mechanistic link between metal exposure and placental dysfunction. Metabolites such as panthenol, tretinoin, and (±)8-HEPE, which showed strong correlations with metal exposure, hold promise for being developed into a panel of circulating biomarkers. Such a panel could aid in the early identification of at-risk women, improve RPL risk stratification, and provide a more objective measure of the biological impact of environmental exposures.

### 4.4 Study strengths and limitations

This pioneering study integrates metabolomics with mixed-metal exposure analysis in RPL to elucidate underlying biological mechanisms. Employing an innovative framework, we combined metal and metabolic biomarkers, using machine learning to identify key drivers of RPL while accounting for complex variable interactions. The BKMR model provided robust exposure-response estimations for dominant metals, and Qgcomp yielded parametric mixture effect estimates. However, our study does have several limitations. First, the analytic data came from peripheral blood samples may not fully capture the biochemical environment within the uterus. The detected metabolites in the blood may not necessarily correspond to those in the reproductive system levels. However, recent research has shown significant correlations between blood and reproductive metabolite levels. Third, the cross-sectional study design does not allow for detecting the dynamic fluctuations of metabolic profiles over time, potentially impacting the interpretation of the results. Fourth, although this study employed an untargeted metabolomic approach, identifying detected peaks was still contingent upon comparison with an existing metabolomic library.

Overall, our study provides valuable insights into the metabolic and environmental factors associated with RPL. The findings emphasize the importance of considering both intrinsic metabolic alterations and extrinsic environmental exposures in understanding and managing RPL risk. Future research should focus on validating these biomarkers in larger cohorts and exploring the underlying mechanisms through which they influence pregnancy outcomes. Additionally, interventions aimed at improving metabolic health could be beneficial for women at risk of RPL.

## 5 Conclusion

Metals exposure is linked to a higher risk of RPL, possibly through the dysregulation of purine metabolism, pantothenate and CoA biosynthesis, retinol metabolism and ubiquinone/terpenoid-quinone biosynthesis. Larger, prospective studies are needed to confirm these findings.

## Data Availability

The raw data supporting the conclusions of this article will be made available by the authors, without undue reservation.

## References

[1] ESHRE Guideline Group on RPL Bender Atik R Christiansen OB Elson J Kolte AM Lewis S . ESHRE guideline: recurrent pregnancy loss: an update in 2022. Hum Reprod Open. (2023) 2023:hoad002. doi: 10.1093/hropen/hoad002, PMID: 36873081 PMC9982362

[2] Dimitriadis E Menkhorst E Saito S Kutteh WH Brosens JJ . Recurrent pregnancy loss. Nat Rev Dis Primers. (2020) 6:98. doi: 10.1038/s41572-020-00228-z, PMID: 33303732

[3] Bedaiwy MA Fayek B Yang EC Iews MS Elgendi M Abdelkareem AO . Prevalence, causes, and impact of non-visualized pregnancy losses in a recurrent pregnancy loss population. Hum Reprod. (2023) 38:830–9. doi: 10.1093/humrep/dead040, PMID: 36881694

[4] Kolte AM Olsen LR Mikkelsen EM Christiansen OB Nielsen HS . Depression and emotional stress is highly prevalent among women with recurrent pregnancy loss. Hum Reprod. (2015) 30:777–82. doi: 10.1093/humrep/dev014, PMID: 25662810 PMC4359400

[5] Monticciolo I Guarano A Inversetti A Barbaro G Di Simone N . Unexplained recurrent pregnancy loss: clinical application of immunophenotyping. Am J Reprod Immunol. (2024) 92:e13939. doi: 10.1111/aji.13939, PMID: 39392245

[6] Ren M Wang L Wen L Chen J Quan S Shi X . Association between female circulating heavy metal concentration and abortion: a systematic review and meta-analysis. Front Endocrinol (Lausanne). (2023) 14:1216507. doi: 10.3389/fendo.2023.1216507, PMID: 37711903 PMC10497972

[7] LankaduraiBrian P NagatoEdward G SimpsonMyrna J . Environmental metabolomics: an emerging approach to study organism responses to environmental stressors. Environ Rev. (2013) 21:180–205. doi: 10.1139/er-2013-0011

[8] Bundy JG Davey MP Viant MR . Environmental metabolomics: a critical review and future perspectives. Metabolomics. (2009) 5:3–21. doi: 10.1007/s11306-008-0152-0

[9] Shi Y Li K Ding R Li X Cheng Z Liu J . Untargeted metabolomics and machine learning unveil the exposome and metabolism linked with the risk of early pregnancy loss. J Hazard Mater. (2025) 488:137362. doi: 10.1016/j.jhazmat.2025.137362, PMID: 39892135

[10] Yang X Mu F Zhang J Yuan L Zhang W Yang Y . Reproductive factors and subsequent pregnancy outcomes in patients with prior pregnancy loss. BMC Pregnancy Childbirth. (2024) 24:219. doi: 10.1186/s12884-024-06422-1, PMID: 38528474 PMC10964557

[11] Ding N Yang X Wang R Wang F . Metabolomics profiling identifies diagnostic metabolic signatures for pregnancy loss: a cross-sectional study from northwestern China. Front Endocrinol (Lausanne). (2025) 16:1518043. doi: 10.3389/fendo.2025.1518043, PMID: 40276553 PMC12018233

[12] Akoglu H . User’s guide to correlation coefficients. Turk J Emerg Med. (2018) 18:91–3. doi: 10.1016/j.tjem.2018.08.001, PMID: 30191186 PMC6107969

[13] Keil AP Buckley JP O’Brien KM Ferguson KK Zhao S White AJ . A quantile-based g-computation approach to addressing the effects of exposure mixtures. Environ Health Perspect. (2020) 128:47004. doi: 10.1289/EHP5838, PMID: 32255670 PMC7228100

[14] Bobb JF Birgit LV Henn C David C Christiani RO Wright MM . Bayesian kernel machine regression for estimating the health effects of multi-pollutant mixtures. Biostatistics. (2015) 16:493–508. doi: 10.1093/biostatistics/kxu058, PMID: 25532525 PMC5963470

[15] Zhang Y Yan X Tan J Tan J Liu C Yang P . Exposure of reproductive-aged women to multiple metals and its associations with unexplained recurrent miscarriage. Toxics. (2023) 11:830. doi: 10.3390/toxics11100830, PMID: 37888681 PMC10611235

[16] Zhang X Wei H Guan Q Yang X Yu Q Zhang M . Maternal exposure to trace elements, toxic metals, and longitudinal changes in infancy anthropometry and growth trajectories: A prospective cohort study. Environ Sci Technol. (2023) 57:11779–91. doi: 10.1021/acs.est.3c02535, PMID: 37525382

[17] Gonzalez-Martin R Palomar A Perez-Deben S Salsano S Quiñonero A Caracena L . Associations between non-essential trace elements in women’s biofluids and IVF outcomes in euploid single-embryo transfer cycles. J Xenobiot. (2024) 14:1093–108. doi: 10.3390/jox14030062, PMID: 39189177 PMC11348048

[18] Cornu R Béduneau A Martin H . Ingestion of titanium dioxide nanoparticles: a definite health risk for consumers and their progeny. Arch Toxicol. (2022) 96:2655–86. doi: 10.1007/s00204-022-03334-x, PMID: 35895099

[19] Karimipour M Zirak Javanmard M Ahmadi A Jafari A . Oral administration of titanium dioxide nanoparticle through ovarian tissue alterations impairs mice embryonic development. Int J Reprod Biomed. (2018) 16:397–404. doi: 10.29252/ijrm.16.6.397, PMID: 30123868 PMC6079309

[20] Wu Y Chen L Chen F Zou H Wang Z . A key moment for TiO2: Prenatal exposure to TiO2 nanoparticles may inhibit the development of offspring. Ecotoxicol Environ Saf. (2020) 202:110911. doi: 10.1016/j.ecoenv.2020.110911, PMID: 32800246

[21] Dugershaw-Kurzer B Bossart J Buljan M Hannig Y Zehnder S Gupta G . Nanoparticles dysregulate the human placental secretome with consequences on angiogenesis and vascularization. Adv Sci (Weinh). (2024) 11:e2401060. doi: 10.1002/advs.202401060, PMID: 38767187 PMC11267331

[22] D’Errico JN Doherty C Reyes George JJ Buckley B Stapleton PA . Maternal, placental, and fetal distribution of titanium after repeated titanium dioxide nanoparticle inhalation through pregnancy. Placenta. (2022) 121:99–108. doi: 10.1016/j.placenta.2022.03.008, PMID: 35305398 PMC9010360

[23] Zhang Q Sun X Xiao X Zheng J Li M Yu M . The effect of maternal chromium status on lipid metabolism in female elderly mice offspring and involved molecular mechanism. Biosci Rep. (2017) 37:BSR20160362. doi: 10.1042/BSR20160362, PMID: 28320771 PMC5408666

[24] Yao X Huang S Li Y Ge Y Zhang Z Ning J . Transgenerational effects of zinc, selenium and chromium supplementation on glucose homeostasis in female offspring of gestational diabetes rats. J Nutr Biochem. (2022) 110:109131. doi: 10.1016/j.jnutbio.2022.109131, PMID: 36028097

[25] Cicero AFG Fogacci F Di Micoli V Angeloni C Giovannini M Borghi C . Purine metabolism dysfunctions: experimental methods of detection and diagnostic potential. Int J Mol Sci. (2023) 24:7027. doi: 10.3390/ijms24087027, PMID: 37108190 PMC10138451

[26] Huang Z Xie N Illes P Di Virgilio F Ulrich H Semyanov A . From purines to purinergic signalling: molecular functions and human diseases. Signal Transduct Target Ther. (2021) 6:162. doi: 10.1038/s41392-021-00553-z, PMID: 33907179 PMC8079716

[27] Ma T Liu T Xie P Jiang S Yi W Dai P Guo X . UPLC-MS-based urine nontargeted metabolic profiling identifies dysregulation of pantothenate and CoA biosynthesis pathway in diabetic kidney disease. Life Sci. (2020) 258:118160. doi: 10.1016/j.lfs.2020.118160, PMID: 32730837

[28] Slyshenkov VS Rakowska M Wojtczak L . Protective effect of pantothenic acid and related compounds against permeabilization of Ehrlich ascites tumour cells by digitonin. Acta Biochim Pol. (1996) 43:407–10. doi: 10.18388/abp.1996_4512, PMID: 8862188

[29] Depeint F Bruce WR Shangari N Mehta R O’Brien PJ . Mitochondrial function and toxicity: role of B vitamins on the one-carbon transfer pathways. Chem Biol Interact. (2006) 163:113–32. doi: 10.1016/j.cbi.2006.05.010, PMID: 16814759

[30] Tay YJ Liang J Yao S Han M . Integrated Transcriptomic Analysis Uncovers the Protective Effects of Berberine Hydrochloride in Enhancing Hepatic Caecum Response of Branchiostoma belcheri (Chinese lancelet) to Aeromonas hydrophila. Mar Biotechnol (NY). (2025) 27:100. doi: 10.1007/s10126-025-10469-2, PMID: 40549185

[31] Ren X Tian B Wang L Tan Y Huang Y Jiang X Liu Y . Metabolomics integrated with transcriptomics reveals the changes during developmental stages in Shiraia bambusicola. J Basic Microbiol. (2022) 62:721–39. doi: 10.1002/jobm.202200008, PMID: 35289436

[32] Hussain T Murtaza G Metwally E Kalhoro DH Kalhoro MS Rahu BA . The role of oxidative stress and antioxidant balance in pregnancy. Mediators Inflamm. (2021) 2021:9962860. doi: 10.1155/2021/9962860, PMID: 34616234 PMC8490076

[33] Panelli DM Bianco K . Cellular aging and telomere dynamics in pregnancy. Curr Opin Obstet Gynecol. (2022) 34:57–61. doi: 10.1097/GCO.0000000000000765, PMID: 34845136 PMC8891073

[34] Miroux-Catarino A Silva L Amaro C Viana I . Allergic contact dermatitis caused dexpanthenol-But is that all? Contact Dermatitis. (2019) 81:391–2. doi: 10.1111/cod.13341, PMID: 31231804

[35] Weber B Hylwa S . Panthenol allergic contact dermatitis: sources of exposure, reported cases, and a call for more frequent testing. Dermatitis. (2024) 36:343–351. doi: 10.1089/derm.2024.0489, PMID: 39714944

[36] Saito M Ishida N Yamada H Ibi M Hirose M . 8-HEPE-concentrated materials from pacific krill improve plasma cholesterol levels and hepatic steatosis in high cholesterol diet-fed low-density lipoprotein (LDL) receptor-deficient mice. Biol Pharm Bull. (2020) 43:919–24. doi: 10.1248/bpb.b20-00162, PMID: 32475913

[37] Pal A Sun S Armstrong M Manke J Reisdorph N Adams VR . Beneficial effects of eicosapentaenoic acid on the metabolic profile of obese female mice entails upregulation of HEPEs and increased abundance of enteric Akkermansia muciniphila. Biochim Biophys Acta Mol Cell Biol Lipids. (2022) 1867:159059. doi: 10.1016/j.bbalip.2021.159059, PMID: 34619367 PMC8627244

[38] Zhao Q Samuels CA Timmins P Massri N Chemerinski A Wu T . Signaling via retinoic acid receptors mediates decidual angiogenesis in mice and human stromal cell decidualization. FASEB J. (2025) 39:e70291. doi: 10.1096/fj.202400766R, PMID: 39777800 PMC11706222

[39] Yu J Berga SL Zou W . Human endometrial stromal cell differentiation is stimulated by PPARbeta/delta activation: new targets for infertility? J Clin Endocrinol Metab. (2020) 105:2983–95. doi: 10.1210/clinem/dgaa413, PMID: 32594141 PMC7373326

[40] Gou Y Liu JJ Zhang JF Yang WP Yang JZ Feng K . Identifying biomarkers distinguishing sepsis after trauma from trauma-induced SIRS based on metabolomics data: a retrospective study. Sci Rep. (2025) 15:13748. doi: 10.1038/s41598-025-94701-y, PMID: 40258847 PMC12012006

