# Supplementary material for: Linking the metals to metabolism in recurrent pregnancy loss through untargeted metabolomics and machine learning
Source: Front Endocrinol (Lausanne). 2025 Dec 8;16:1679190. doi: 10.3389/fendo.2025.1679190 (PMC12719250; doi:10.3389/fendo.2025.1679190)
Supplement: Supplementary file 1 [file DataSheet1.docx]

**Linking the Metals to Metabolism in Recurrent Pregnancy Loss through Untargeted Metabolomics and Machine Learning**

**STable 1.** Lower detection of the metals in our study........................................................................................................................................ .2

**STable 2.**Profiling of 12 elements in serum of case–control group.....................................................................................................................3

**S Figure 1.**The AUC curve based on five MLalgorithms....................................................................................................................................4

**S Table 3.** Characteristics of participants in this study for integrated metals and untargeted metabolism. ...................................................5

**S Table 4.**The spearman relationship between Variables and Metals .................................................................................................................6

**S Table 5.**The spearman relationship between Variables and Metabolites.......................................................................................................... 7

**S Table 6.**The spearman relationship between Li and Metabolite........................................................................................................................7

**S Table 7.** The spearman relationship between V and Metabolites.................................................................................................................... 15

**S Table 8.**The spearman relationship between Ti and Metabolites..................................................................................................................... 22

**S Table 9.** The spearman relationship between Cr and Metabolites.....................................................................................................................25

**S Table 10.**The spearman relationship between Ni and Metabolites....................................................................................................................27

**S Figure 2.** KEGG for the significant associated metabolites with five heavy metals........................................................................................ 32

**S Figure 3.** The correlations between metals and metabolites.............................................................................................................................34

**Table S1.** Lower detection of the metals in our study

| Metals | LOD |
| --- | --- |
| Li (ug/L) | 0.2 |
| Al (ug/L) | 100 |
| Ti (ug/L) | 0.2 |
| Sr (ug/L) | 0.2 |
| Cd (ug/L) | 0.2 |
| Tl ( ug/L) | 1 |
| Pb (ug/L) | 4 |
| V (ug/L) | 0.2 |
| Cr (ug/L) | 0.2 |
| Co (ug/L) | 0.2 |
| Ni (ug/L) | 2 |
| Cu (ug/L) | 100 |
| Zn (ug/L) | 100 |
| Fe (ug/L) | 100 |
| As (ug/L) | 2 |
| Se (ug/L) | 2 |

**S Table 2. Profiling of 12 elements in serum of case–control group.**

| Metals | Total(644) | RPL(318) | Controls(326) |
| --- | --- | --- | --- |
| Li (ug/L) | 4.79(2.36-10.69) | 2.35(1.75-3.25) | 10.35(8.31-11.80) |
| Al (ug/L) | 502.44(272.47-916.46) | 470.07(288.87-815.92) | 578.55(247.98-1002.67) |
| Ti (ug/L) | 123.52(104.04-152.91) | 143.77(120.58-173.01) | 107.99(77.08-128.83) |
| Sr (ug/L) | 83.73(63.11-114.53) | 80.16(58.97-109.87) | 90.38(66.92-117.42) |
| Pb (ug/L) | 5.69(2.83-8.81) | 5.02(2.83-7.86) | 6.70(4.41-9.92) |
| V (ug/L) | 3.02(2.22-3.80) | 2.22(1.98-2.47) | 3.66(3.25-4.17) |
| Cr (ug/L) | 34.85(21.62-50.40) | 22.18(17.47-33.81) | 47.53(35.03-64.68) |
| Ni (ug/L) | 9.91(2.48-17.72) | 5.14(1.41-14.91) | 12.29(7.40-19.38) |
| Cu (ug/L) | 1006.87(839.40-1227.14) | 1027.62(841.57-1402.49) | 990.04(836.38-1141.93) |
| Zn (ug/L) | 952.75(793.51-1218.90) | 897.81(750.83-1259.73) | 998.03(823.58-1199.96) |
| Fe (ug/L) | 1554.07(1225.61-1980.85) | 1457.54(1139.40-1828.24) | 1675.27(1322.54-2093.02) |
| Se (ug/L) | 111.27(91.26-137.95) | 113.55(94.10-137.99) | 108.79(89.70-137.99) |


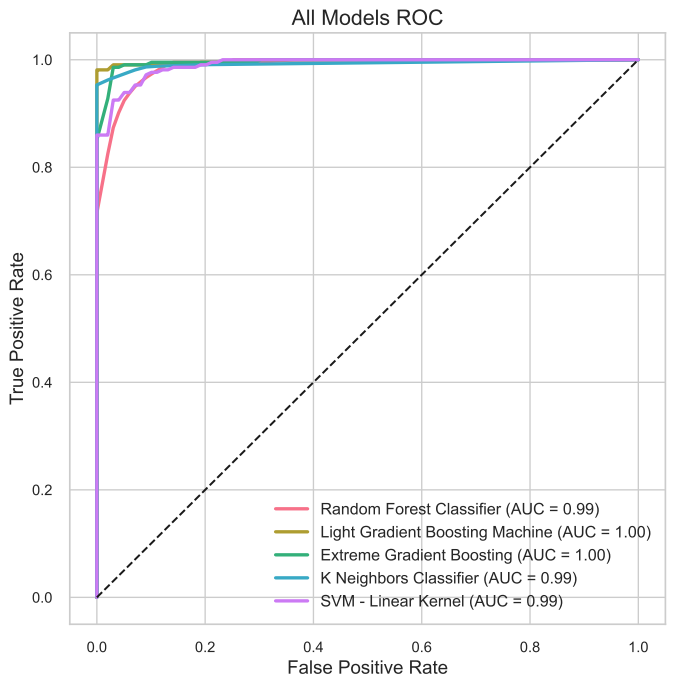


**S Figure 1.The AUC curve based on five MLalgorithms.**

**S Table 3. Characteristics of participants in this study for integrated metals and untargeted metabolism.**

| Variables | Pregnancy loss | Control | *P* |
| --- | --- | --- | --- |
| n | 50 | 50 | *-* |
| Age | 30.38±3.23 | 31.12±3.04 | 0.241 |
| BMI | 22.86±2.87 | 22.29±4.10 | 0.424 |
| SBP | 114.50±13.80 | 110.10±10.12 | 0.072 |
| DBP | 72.44±10.14 | 71.18±8.71 | 0.507 |
| WHR | 0.86±0.06 | 0.82±0.05 | <0.001 |
| Education  ≤ High school  ˃ High school |  |  | <0.001 |
|  | 17(34.0%) | 3(6.0%) |  |
|  | 33(66.0%) | 47(94.0%) |  |
| Ethnicity |  |  | 0.242 |
| Chinese Han | 50 | 0 |  |
| Minority | 47 | 3 |  |

**S Table 4.The spearman relationship between Variables and Metals.**

| Variables | Li | Al | Ti | Sr | Pb | V | Cr | Ni | Cu | Zn | Fe | Se |
| --- | --- | --- | --- | --- | --- | --- | --- | --- | --- | --- | --- | --- |
| age | 0.245 | 0.115 | -0.16 | -0.062 | 0.144 | 0.199 | 0.201 | 0.102 | -0.125 | -0.049 | 0.024 | -0.088 |
| Education | -0.204 | -0.062 | 0.077 | -0.038 | -0.048 | -0.269 | -0.228 | -0.132 | -0.041 | -0.097 | -0.097 | -0.180 |
| Ethiniciity | -0.062 | -0.078 | 0.004 | -0.024 | -0.079 | -0.035 | -0.062 | -0.052 | 0.092 | 0.006 | -0.025 | 0.025 |
| BMI | -0.010 | -0.057 | -0.026 | -0.016 | -0.023 | 0.018 | -0.056 | -0.050 | 0.122 | 0.027 | -0.029 | 0.037 |
| WHR | -0.269 | 0.097 | 0.212 | 0.052 | -0.005 | -0.178 | -0.112 | -0.113 | 0.119 | -0.003 | -0.057 | 0.052 |

**S Table 5.The spearman relationship between Variables and Metabolites.**

| Variables | Panthenol | (+/-)-8-HEPE | (+/-)-Cannabichromeorcin | 7-alpha-carboxy-17-alpha-carboxyethylandrostan lactone phenyl ester | Tretinoin |
| --- | --- | --- | --- | --- | --- |
| age | .165 | 0.169 | 0.117 | 0.015 | 0.191 |
| Education | -.008 | 0.241 | 0.267 | 0.239 | 0.180 |
| Ethnicity | .054 | -0.049 | -0.081 | -0.024 | -0.055 |
| BMI | .044 | 0.055 | 0.104 | 0.019 | 0.004 |
| WHR | .086 | 0-.214 | 0-.184 | -0.225 | -0.162 |

**S Table 6.The spearman relationship between Li and Metabolite**

| Metabolites | r |
| --- | --- |
| Calcitriol | .197 |
| 8-{[(3S)-3-(1H-Benzimidazol-2-yl)-1-pyrrolidinyl]sulfonyl}quinoline | .200 |
| 6-(3-hydroxybutan-2-yl)-5-(hydroxymethyl)-4-methoxy-2H-pyran-2-one | .201 |
| N-Stearoyl taurine | .202 |
| D-Panthenol | .202 |
| Atropine | -.202 |
| Hex3Cer 23:2;2O | -.202 |
| Stachyose | -.203 |
| 1,7,8-trihydroxy-3-methyl-1,2,3,4,7,12-hexahydrotetraphen-12-one | .207 |
| N-{5-[(dimethylamino)sulfonyl]-2-methylphenyl}cyclohexanecarboxamide | -.208 |
| 1-[4-(1-adamantyl)phenoxy]-3-piperidinopropan-2-ol hydrochloride | .209 |
| AKB48 N-(4-hydroxypentyl) metabolite | -.209 |
| DL-Arginine | -.210 |
| Paliperidone | -.210 |
| MAM2201 N-pentanoic acid metabolite | -.212 |
| Palmitoylethanolamide | .214 |
| QLK | -.214 |
| (5S)-5-hydroxy-1,7-diphenylheptan-3-one | .215 |
| NVP-231 | -.215 |
| Virginiamycin | -.216 |
| RQH | -.216 |
| PC(16:0/16:0) | .217 |
| 11(E)-Eicosenoic Acid | .217 |
| 2-[2-(1-isobutylcyclohexyl)-1-methylethylidene]hydrazine-1-carboxamide | -.218 |
| 3-(1H-benzo[d]imidazol-2-yl)-6-ethyl-7-hydroxy-4H-chromen-4-one | -.218 |
| MGDG O-13:1_4:0 | -.218 |
| Toltrazuril | -.219 |
| TKK | -.220 |
| N1-(4-chlorophenyl)-3-(1H-pyrrol-1-ylmethyl)piperidine-1-carboxamide | -.221 |
| D-(-)-Quinic acid | .223 |
| 20-Hydroxy-(5Z,8Z,11Z,14Z)-eicosatetraenoic acid | .224 |
| 1,7-bis(4-hydroxyphenyl)-5-methoxyheptan-3-one | -.224 |
| DG O-18:3_16:2 | .225 |
| IPH | -.225 |
| CAR 6:2 | -.226 |
| 1-morpholino-3-(4-nitrophenoxy)propan-2-ol | -.227 |
| Decanoylcarnitine | -.227 |
| 13,14-dihydro-15-keto Prostaglandin A2 | .228 |
| YMK | -.228 |
| Triethanolamine | .229 |
| RLK | -.229 |
| Adenylocuccinic Acid | -.230 |
| Acetylcysteine | -.232 |
| Elaidic acid | .233 |
| Oleoyl ethylamide | .234 |
| N'-[(2,4-dihydroxyphenyl)methylene]-4-methylbenzenesulfonohydrazide | -.235 |
| N~5~-(pyridin-2-ylmethyl)-1H-1,2,4-triazole-3,5-diamine | .237 |
| Pantetheine | -.237 |
| CAR 8:0 | -.237 |
| JWH 250 N-pentanoic acid metabolite | -.238 |
| N1-Acetylspermine | -.238 |
| CAR 15:0 | -.239 |
| FPH | -.240 |
| Palmitoleic Acid | .241 |
| N-Formylkynurenine | .241 |
| 5-chloro-N-(4-morpholinophenyl)-1H-indole-2-carboxamide | -.241 |
| LPC 10:0-SN1 | .242 |
| (+/-)9(10)-EpOME | .244 |
| 11(Z),14(Z),17(Z)-Eicosatrienoic acid | .245 |
| Solanine | -.245 |
| FAHFA 4:0/24:4 | .248 |
| α-Lactose | .248 |
| L(-)-Carnitine | -.248 |
| FAHFA 4:0/18:1 | .249 |
| Bialaphos | -.250 |
| 5-[(E)-2-(3,5-dihydroxyphenyl)ethenyl]-2-methoxybenzene-1,3-diol | -.251 |
| Indole-3-butyric acid | .253 |
| N'2-benzylidene-5-hex-1-ynylfuran-2-carbohydrazide | -.256 |
| Tridecylic acid | -.257 |
| LPA 15:0 | .258 |
| Desoxycortone | .258 |
| Prostaglandin H1 | -.258 |
| L-cysteine | -.259 |
| Cer 18:0;2O/24:0 | -.259 |
| 2-acetamido-3-(4-methoxyphenyl)propanoic acid | -.260 |
| (±)9-HpODE | .263 |
| FKK | -.263 |
| 1-acetyl-N-(6-chloro-1,3-benzothiazol-2-yl)-4-piperidinecarboxamide | -.265 |
| CAR 7:0 | -.267 |
| 3-[(5-nitropyridin-2-yl)oxy]-1H-indazole | -.267 |
| Lysopa 18:0 | .268 |
| 1-(4-bromophenyl)-2-phenylethan-1-one | .268 |
| 1,3,7-trimethyl-2,3,6,7-tetrahydro-1H-purine-2,6-dione | -.270 |
| IPK | -.271 |
| Adenosine 5'-Diphosphate | -.271 |
| 3-Acetyl-11-keto-β-boswellic acid | .272 |
| N-Desmethyltramadol | .273 |
| Lysopc 20:0 | .274 |
| LPK | -.274 |
| Lysopa 16:0 | .275 |
| Fenpropimorph | .279 |
| 2-(Dimethylamino)Guanosine | .279 |
| Diethyl phosphate | -.279 |
| N1-(1-ethyl-2-oxo-1,2-dihydrobenzo[cd]indol-6-yl)-3-nitrobenzamide | -.279 |
| Dihydromethysticin | -.282 |
| Phylloquinone | .283 |
| Eicosapentaenoic acid | .285 |
| QNK | -.285 |
| QPH | -.287 |
| Oxoamide | .289 |
| 2-[(3S)-1-Benzyl-3-pyrrolidinyl]-5-(trifluoromethyl)-1H-benzimidazole | -.297 |
| Melibiose | .302 |
| 2-Methoxyestradiol (2-MeOE2) | .304 |
| Ursolic acid | .304 |
| Dopamine | -.304 |
| LPS 20:3 | .305 |
| L-Glutathione oxidized | .306 |
| trans-Zeatin | .308 |
| Oleamide | .310 |
| 2-(14,15-Epoxyeicosatrienoyl) glycerol | .312 |
| All trans-Retinal | .313 |
| RMH | -.313 |
| C-6 NBD ceramide | -.313 |
| 4'-Methoxyacetophenone | .315 |
| 2-Arachidonoyl glycerol | .319 |
| 5-[(10Z)-14-(3,5-dihydroxyphenyl)tetradec-10-en-1-yl]benzene-1,3-diol | .319 |
| Quinine | .320 |
| ST 24:2;O4 | .322 |
| KNK | -.322 |
| Cefdinir | -.322 |
| LysoPC 12:1 | -.325 |
| N-(4-fluorophenyl)-2-[4-(5-propyl-2-pyrimidinyl)piperazino]acetamide | -.325 |
| 3-(2-methylpropyl)-octahydropyrrolo[1,2-a]pyrazine-1,4-dione | .326 |
| YPH | -.326 |
| Hydrocortisone acetate | .327 |
| 18-β-Glycyrrhetinic acid | .329 |
| Thromboxane B2-biotin | -.331 |
| 2-hydroxy-6-[(8Z,11Z)-pentadeca-8,11,14-trien-1-yl]benzoic acid | .332 |
| Cytidine-5'-monophosphate | -.333 |
| 8-iso Prostaglandin A2 | .336 |
| 4-Guanidinobutyric acid | .338 |
| UMP | -.340 |
| 3-Coumaric acid | .342 |
| Ala-trp | -.343 |
| 1-Methylguanosine | -.344 |
| N-Tetradecanamide | .345 |
| Inosine | -.347 |
| Guanosine-3',5'-cyclic monophosphate | -.351 |
| (2E)-4-Hydroxy-3,7-dimethyl-2,6-octadien-1-yl beta-D-glucopyranoside | -.351 |
| THC | .354 |
| 7-Ketolithocholic acid | .363 |
| Diphenylamine | -.363 |
| 2,4-Dimethylbenzaldehyde | .370 |
| Guanosine | -.371 |
| Guanine | -.377 |
| Adenosine | -.393 |
| LPG O-18:2 | -.396 |
| 7-alpha-carboxy-17-alpha-carboxyethylandrostan lactone phenyl ester | .405 |
| Dipropylene glycol dimethyl ether | -.418 |
| Tretinoin | .436 |
| (+/-)-Cannabichromeorcin | .455 |
| (+/-)8-HEPE | .486 |
| Panthenol | -.512 |

**S Table 7. The spearman relationship between V and Metabolites**

| Metabolites | r |
| --- | --- |
| ST 28:1;O;Hex;FA 18:2 | .197 |
| N~5~-(pyridin-2-ylmethyl)-1H-1,2,4-triazole-3,5-diamine | .202 |
| Pregnanetriol | .204 |
| 5-[(E)-2-(3,5-dihydroxyphenyl)ethenyl]-2-methoxybenzene-1,3-diol | -.206 |
| WQH | -.206 |
| FAHFA 5:0/20:2 | .207 |
| all-cis-4,7,10,13,16-Docosapentaenoic acid | .207 |
| N-Acetylanthranilic acid | -.207 |
| Trenbolone acetate | -.208 |
| 8-{[(3S)-3-(1H-Benzimidazol-2-yl)-1-pyrrolidinyl]sulfonyl}quinoline | .209 |
| Hexadecanamide | .209 |
| FAHFA 6:0/20:2 | .209 |
| Cefdinir | -.209 |
| CAR 6:2 | -.210 |
| 4-(tert-butyl)phenyl 3,5-dimethylisoxazole-4-carboxylate | -.210 |
| Arachidonic acid | .211 |
| Acetylcysteine | -.211 |
| (+/-)9,10-dihydroxy-12Z-octadecenoic acid | -.211 |
| 2-[2-(2-pyridyloxy)ethoxy]pyridine | .213 |
| Adenosine 5'-Diphosphate | -.213 |
| Bialaphos | -.214 |
| MGDG O-13:1_4:0 | -.215 |
| 1,7,8-trihydroxy-3-methyl-1,2,3,4,7,12-hexahydrotetraphen-12-one | .216 |
| TKK | -.217 |
| HBMP 18:2_18:2_12:0 | .218 |
| N-Stearoyl taurine | .218 |
| methyl 2-(acetylamino)-4,5-dimethoxybenzoate | -.219 |
| Adenylocuccinic Acid | -.221 |
| N1-(1-ethyl-2-oxo-1,2-dihydrobenzo[cd]indol-6-yl)-3-nitrobenzamide | -.221 |
| Oleoyl ethylamide | .222 |
| 3-(2-methylpropyl)-octahydropyrrolo[1,2-a]pyrazine-1,4-dione | .223 |
| 13,14-dihydro-15-keto-tetranor Prostaglandin E2 | .224 |
| FAHFA 4:0/18:0 | .228 |
| Inosine | -.230 |
| 1,7-bis(4-hydroxyphenyl)-5-methoxyheptan-3-one | -.230 |
| 1-Methylguanosine | -.232 |
| 2-Methoxyestradiol (2-MeOE2) | .233 |
| LPA 15:0 | .234 |
| SM 8:0;2O/19:0 | .234 |
| LPC 10:0-SN1 | .236 |
| Hydrocortisone acetate | .237 |
| YMK | -.239 |
| Guanine | -.240 |
| N1-Acetylspermine | -.240 |
| PC(16:0/16:0) | .242 |
| (+/-)9(10)-EpOME | .244 |
| Stachyose | -.244 |
| Palmitoylethanolamide | .247 |
| Desoxycortone | .249 |
| 1-acetyl-N-(6-chloro-1,3-benzothiazol-2-yl)-4-piperidinecarboxamide | -.249 |
| N'-[(2,4-dihydroxyphenyl)methylene]-4-methylbenzenesulfonohydrazide | -.249 |
| 2-(14,15-Epoxyeicosatrienoyl) glycerol | .252 |
| (+/-)13-HODE | .252 |
| N1-(4-chlorophenyl)-3-(1H-pyrrol-1-ylmethyl)piperidine-1-carboxamide | -.252 |
| IPH | -.252 |
| IRH | -.252 |
| DG O-18:3_16:2 | .254 |
| 3-Coumaric acid | .254 |
| trans-Zeatin | .255 |
| 1-(4-bromophenyl)-2-phenylethan-1-one | .256 |
| 1,3,7-trimethyl-2,3,6,7-tetrahydro-1H-purine-2,6-dione | -.256 |
| FAHFA 4:0/20:2 | .257 |
| N1-[6-hydroxy-2-(2-pyridyl)pyrimidin-4-yl]acetamide | .257 |
| MAM2201 N-pentanoic acid metabolite | -.258 |
| Oxoamide | .259 |
| Guanosine | -.259 |
| NVP-231 | -.259 |
| 2-acetamido-3-(4-methoxyphenyl)propanoic acid | -.259 |
| Decanoylcarnitine | -.259 |
| Atropine | -.260 |
| Paliperidone | -.261 |
| 2,4-Dihydroxybenzoic acid | .262 |
| Quinine | .263 |
| N-Formylkynurenine | .264 |
| CAR 12:3 | -.264 |
| FPH | -.265 |
| Hex3Cer 23:2;2O | -.266 |
| Fenpropimorph | .270 |
| Diethyl phosphate | -.272 |
| FAHFA 18:1/20:3 | .275 |
| N-{5-[(dimethylamino)sulfonyl]-2-methylphenyl}cyclohexanecarboxamide | -.276 |
| CAR 15:0 | -.277 |
| FAHFA 4:0/24:4 | .278 |
| FAHFA 2:0/24:4 | .278 |
| Virginiamycin | -.278 |
| Triethanolamine | .280 |
| QPH | -.285 |
| RMH | -.286 |
| Lysopa 18:0 | .288 |
| Prostaglandin H1 | -.288 |
| 2-[(3S)-1-Benzyl-3-pyrrolidinyl]-5-(trifluoromethyl)-1H-benzimidazole | -.290 |
| (2E)-4-Hydroxy-3,7-dimethyl-2,6-octadien-1-yl beta-D-glucopyranoside | -.292 |
| L(-)-Carnitine | -.292 |
| Oleamide | .293 |
| Calcitriol | .294 |
| QLK | -.294 |
| 3-Acetyl-11-keto-β-boswellic acid | .295 |
| LPS 20:3 | .295 |
| 5-chloro-N-(4-morpholinophenyl)-1H-indole-2-carboxamide | -.299 |
| ST 24:2;O4 | .300 |
| FAHFA 16:0/3:0 | .301 |
| 3-[(5-nitropyridin-2-yl)oxy]-1H-indazole | -.301 |
| 11(E)-Eicosenoic Acid | .302 |
| L-Glutathione oxidized | .302 |
| Lysopc 20:0 | .303 |
| CAR 8:0 | -.303 |
| Ursolic acid | .306 |
| FAHFA 4:0/18:1 | .307 |
| LPS 2:0 | -.307 |
| Lysopa 16:0 | .308 |
| Palmitoleic Acid | .310 |
| (±)9-HpODE | .311 |
| 2-Arachidonoyl glycerol | .311 |
| IKK | -.311 |
| C-6 NBD ceramide | -.311 |
| 11(Z),14(Z),17(Z)-Eicosatrienoic acid | .312 |
| JWH 250 N-pentanoic acid metabolite | -.313 |
| 1-morpholino-3-(4-nitrophenoxy)propan-2-ol | -.318 |
| 18-β-Glycyrrhetinic acid | .319 |
| Pantetheine | -.320 |
| Dopamine | -.323 |
| Cytidine-5'-monophosphate | -.324 |
| QNK | -.324 |
| Quercitrin | -.324 |
| Dihydromethysticin | -.324 |
| Phylloquinone | .325 |
| 2-(Dimethylamino)Guanosine | .325 |
| UMP | -.325 |
| Diphenylamine | -.325 |
| 7-Ketolithocholic acid | .326 |
| 2-[2-(1-isobutylcyclohexyl)-1-methylethylidene]hydrazine-1-carboxamide | -.327 |
| Thromboxane B2-biotin | -.328 |
| 3-(1H-benzo[d]imidazol-2-yl)-6-ethyl-7-hydroxy-4H-chromen-4-one | -.328 |
| Elaidic acid | .329 |
| Tridecylic acid | -.329 |
| Solanine | -.329 |
| LPG O-18:2 | -.332 |
| CAR 7:0 | -.338 |
| N'2-benzylidene-5-hex-1-ynylfuran-2-carbohydrazide | -.343 |
| Dipropylene glycol dimethyl ether | -.349 |
| Guanosine-3',5'-cyclic monophosphate | -.349 |
| 5-[(10Z)-14-(3,5-dihydroxyphenyl)tetradec-10-en-1-yl]benzene-1,3-diol | .350 |
| FKK | -.350 |
| 4-Guanidinobutyric acid | .353 |
| RLK | -.353 |
| KNK | -.354 |
| Adenosine | -.356 |
| 20-Hydroxy-(5Z,8Z,11Z,14Z)-eicosatetraenoic acid | .361 |
| N-Tetradecanamide | .363 |
| 4'-Methoxyacetophenone | .368 |
| LPK | -.369 |
| IPK | -.371 |
| LysoPC 12:1 | -.374 |
| Eicosapentaenoic acid | .380 |
| L-cysteine | -.384 |
| All trans-Retinal | .386 |
| 2,4-Dimethylbenzaldehyde | .390 |
| (5S)-5-hydroxy-1,7-diphenylheptan-3-one | .394 |
| 8-iso Prostaglandin A2 | .397 |
| THC | .400 |
| N-(4-fluorophenyl)-2-[4-(5-propyl-2-pyrimidinyl)piperazino]acetamide | -.402 |
| YPH | -.406 |
| Ala-trp | -.416 |
| 2-hydroxy-6-[(8Z,11Z)-pentadeca-8,11,14-trien-1-yl]benzoic acid | .478 |
| 7-alpha-carboxy-17-alpha-carboxyethylandrostan lactone phenyl ester | .505 |
| Panthenol | -.534 |
| Tretinoin | .537 |
| (+/-)-Cannabichromeorcin | .573 |
| (+/-)8-HEPE | .595 |

**S Table 8.The spearman relationship between Ti and Metabolites.**

| Metabolites | r |
| --- | --- |
| TKK | .197 |
| LPG O-17:2 | -.197 |
| Cefdinir | .198 |
| 13,14-dihydro-15-keto Prostaglandin A2 | -.198 |
| 1,7,8-trihydroxy-3-methyl-1,2,3,4,7,12-hexahydrotetraphen-12-one | -.200 |
| 3-hydroxy-N-(1-hydroxy-4-methylpentan-2-yl)-5-oxo-6-phenylhexanamide | -.200 |
| UMP | .201 |
| (+/-)9(10)-EpOME | -.201 |
| Phylloquinone | -.201 |
| D-δ-Tocopherol | -.201 |
| QPH | .202 |
| 13,14-dihydro-15-keto Prostaglandin D1 | -.205 |
| FAHFA 16:0/3:0 | -.217 |
| 18-β-Glycyrrhetinic acid | -.218 |
| 2,4-Dimethylbenzaldehyde | -.219 |
| α-Lapachone | .221 |
| 4-Guanidinobutyric acid | -.221 |
| L-Glutathione oxidized | -.225 |
| NVP-231 | .230 |
| IRH | .230 |
| N-Desmethyltramadol | -.236 |
| Tetranor-12R-HETE | .238 |
| MAM2201 N-pentanoic acid metabolite | .240 |
| LPE O-18:2 | -.241 |
| 4-decyl-3-hydroxy-5-oxooxolane-2,3-dicarboxylic acid | -.245 |
| TLH | .246 |
| Eicosapentaenoic acid | -.251 |
| FAHFA 20:5/17:2 | -.255 |
| SM 8:0;2O/19:0 | -.257 |
| 4-(tert-butyl)phenyl 3,5-dimethylisoxazole-4-carboxylate | .258 |
| Triethanolamine | -.262 |
| HBMP 18:2_18:2_12:0 | -.263 |
| Palmitoleic Acid | -.267 |
| FKK | .271 |
| RLK | .273 |
| IKK | .276 |
| IPH | .277 |
| ST 28:1;O;Hex;FA 18:2 | -.278 |
| KNK | .288 |
| FAHFA 18:1/20:3 | -.289 |
| 20-Hydroxy-(5Z,8Z,11Z,14Z)-eicosatetraenoic acid | -.293 |
| THC | -.300 |
| FAHFA 20:4/20:4 | -.302 |
| YPH | .314 |
| Guanosine-3',5'-cyclic monophosphate | .330 |
| 5α-Dihydrotestosterone glucuronide | -.342 |
| 2-hydroxy-6-[(8Z,11Z)-pentadeca-8,11,14-trien-1-yl]benzoic acid | -.359 |
| Homotaurine | -.367 |
| (5S)-5-hydroxy-1,7-diphenylheptan-3-one | -.383 |
| Hydrocortisone acetate | -.386 |
| All trans-Retinal | -.394 |
| 8-iso Prostaglandin A2 | -.397 |
| (+/-)-Cannabichromeorcin | -.421 |
| (+/-)8-HEPE | -.429 |
| Tretinoin | -.436 |
| 7-alpha-carboxy-17-alpha-carboxyethylandrostan lactone phenyl ester | -.487 |
| Panthenol | .522 |

**S Table 9. The spearman relationship between Cr and Metabolites.**

| Metabolites | r |
| --- | --- |
| ST 28:1;O;Hex;FA 18:2 | .197 |
| 3-Coumaric acid | .198 |
| Tretinoin | .199 |
| TLH | -.199 |
| (+/-)-Cannabichromeorcin | .203 |
| (+/-)9(10)-EpOME | .204 |
| N-Stearoyl taurine | .204 |
| UMP | -.204 |
| Paliperidone | -.204 |
| 5,7-Dihydroxy-2-(3-hydroxy-4-methoxyphenyl)chroman-4-one | .208 |
| THC | .211 |
| (+/-)8-HEPE | .212 |
| SM 8:0;2O/19:0 | .212 |
| 2-[2-(1-isobutylcyclohexyl)-1-methylethylidene]hydrazine-1-carboxamide | -.212 |
| LPE O-15:1 | .214 |
| Oxoamide | .218 |
| 4-Guanidinobutyric acid | .221 |
| HBMP 18:2_18:2_12:0 | .221 |
| all-cis-4,7,10,13,16-Docosapentaenoic acid | .221 |
| FPH | -.221 |
| 7-Methylguanosine | -.221 |
| 3-[(5-nitropyridin-2-yl)oxy]-1H-indazole | -.222 |
| 2-Arachidonoyl glycerol | .225 |
| LPK | -.228 |
| 4'-Methoxyacetophenone | .229 |
| 7-alpha-carboxy-17-alpha-carboxyethylandrostan lactone phenyl ester | .232 |
| 1,7-bis(4-hydroxyphenyl)-5-methoxyheptan-3-one | -.238 |
| FAHFA 18:1/20:3 | .240 |
| PC(16:0/16:0) | .241 |
| 5α-Dihydrotestosterone glucuronide | .242 |
| Oleoyl ethylamide | .244 |
| DG O-18:3_16:2 | .245 |
| Calcitriol | .246 |
| N1-Acetylspermine | -.249 |
| Fenpropimorph | .250 |
| LPG O-17:2 | .252 |
| IPH | -.253 |
| LPE O-18:2 | .255 |
| FAHFA 20:4/20:4 | .259 |
| Palmitoleic Acid | .260 |
| 1,7,8-trihydroxy-3-methyl-1,2,3,4,7,12-hexahydrotetraphen-12-one | .263 |
| Hydrocortisone acetate | .269 |
| Ergocalciferol | .270 |
| 13,14-dihydro-15-keto-tetranor Prostaglandin E2 | .271 |
| Oleamide | .273 |
| FKK | -.273 |
| 1-[4-(1-adamantyl)phenoxy]-3-piperidinopropan-2-ol hydrochloride | .280 |
| N~5~-(pyridin-2-ylmethyl)-1H-1,2,4-triazole-3,5-diamine | .288 |
| Guanosine-3',5'-cyclic monophosphate | -.290 |
| N-Tetradecanamide | .292 |
| 2,4-Dimethylbenzaldehyde | .295 |
| Homotaurine | .321 |
| Panthenol | -.324 |

**S Table 10.The spearman relationship between Ni and Metabolites.**

| Metabolites | r |
| --- | --- |
| LPE O-15:1 | .198 |
| Toltrazuril | .209 |
| Eicosapentaenoic acid | -.209 |
| 1,7,8-trihydroxy-3-methyl-1,2,3,4,7,12-hexahydrotetraphen-12-one | .215 |
| Homotaurine | .219 |
| Cer 18:0;2O/24:0 | .220 |
| Desoxycortone | -.223 |
| Acetylcysteine | .253 |
| 2-Aminobenzenesulfonamide | .254 |
| N-{5-[(dimethylamino)sulfonyl]-2-methylphenyl}cyclohexanecarboxamide | .283 |
| Obscurolide A1 | .300 |

**A**

**
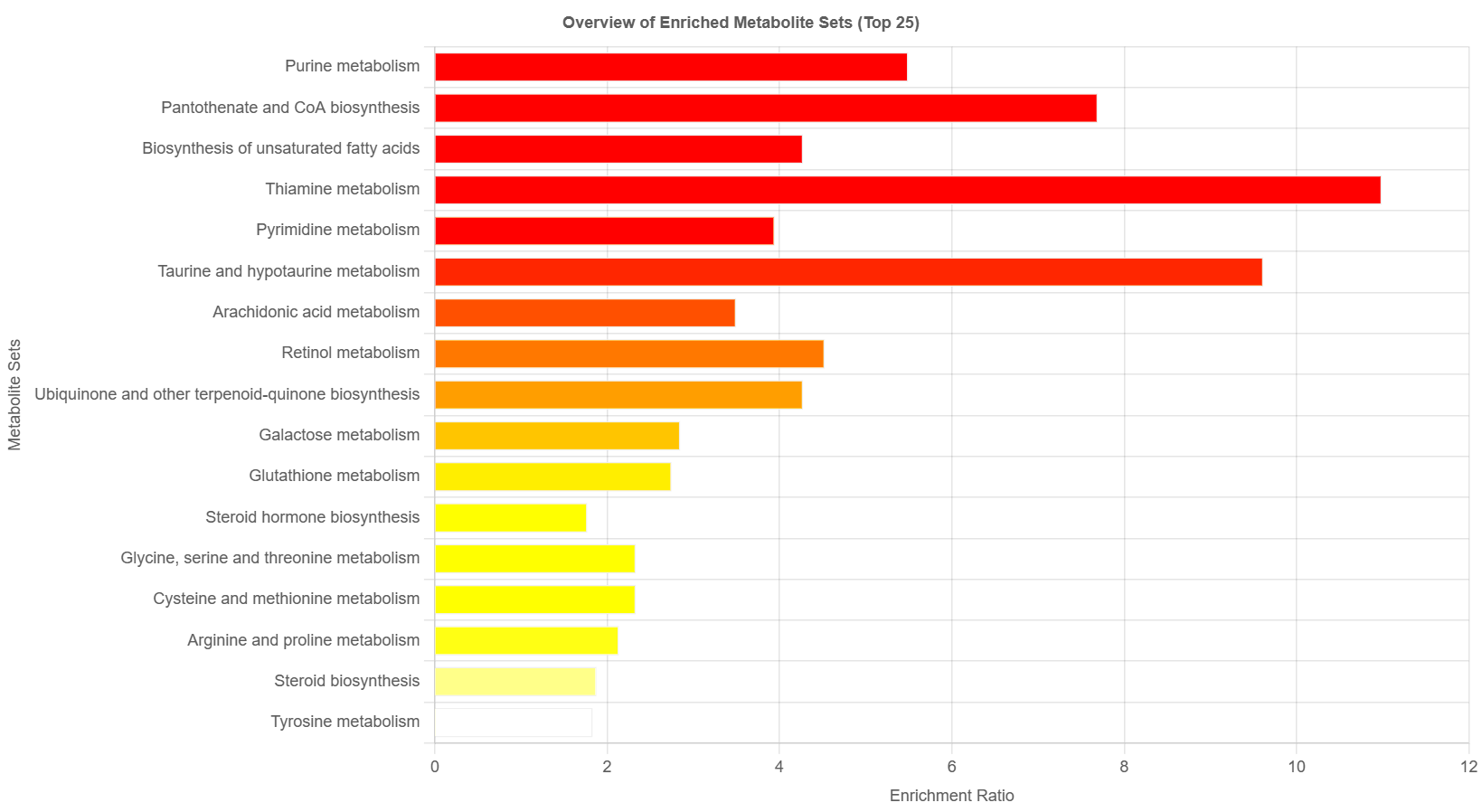
**

**B**

**
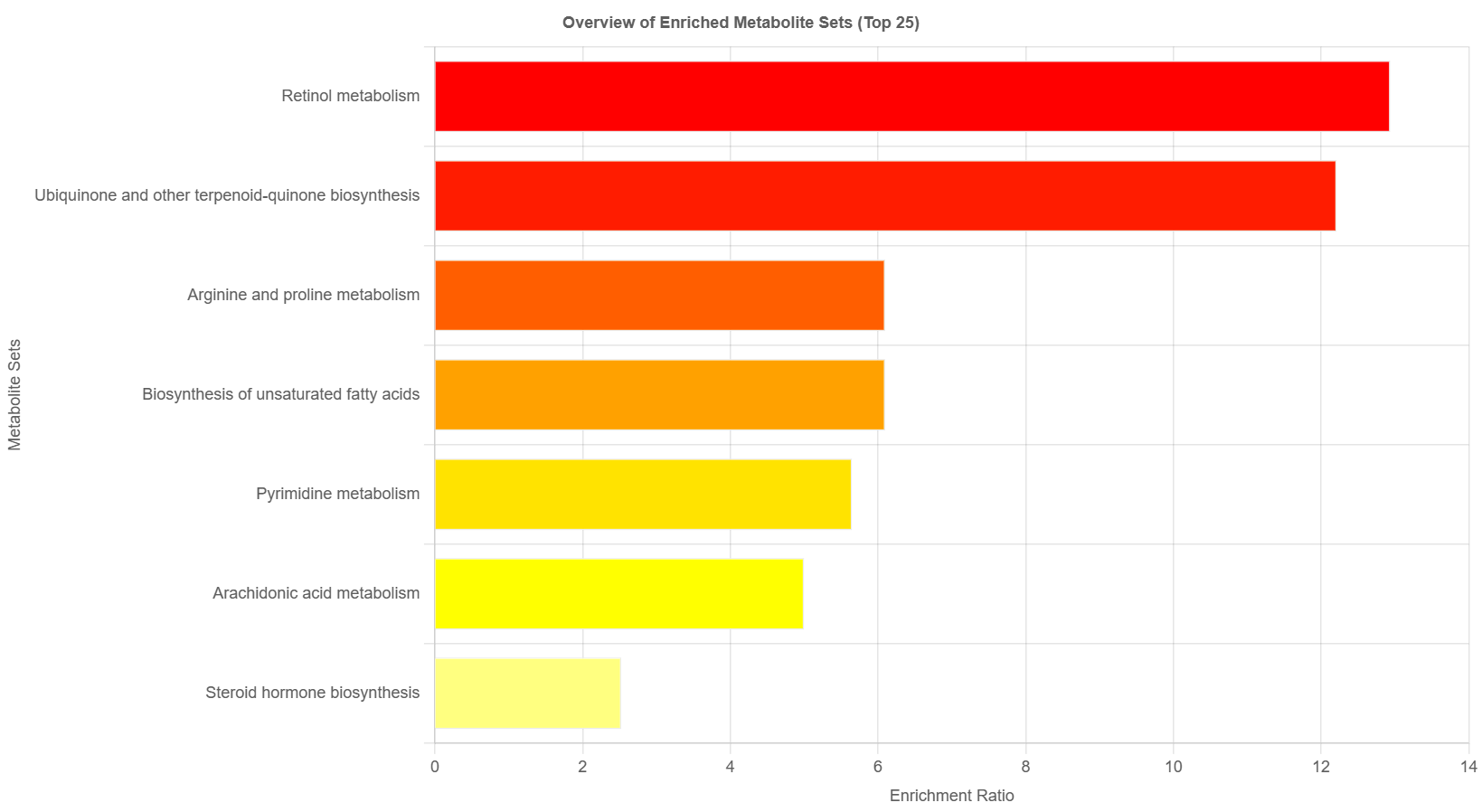
**

**C**

**
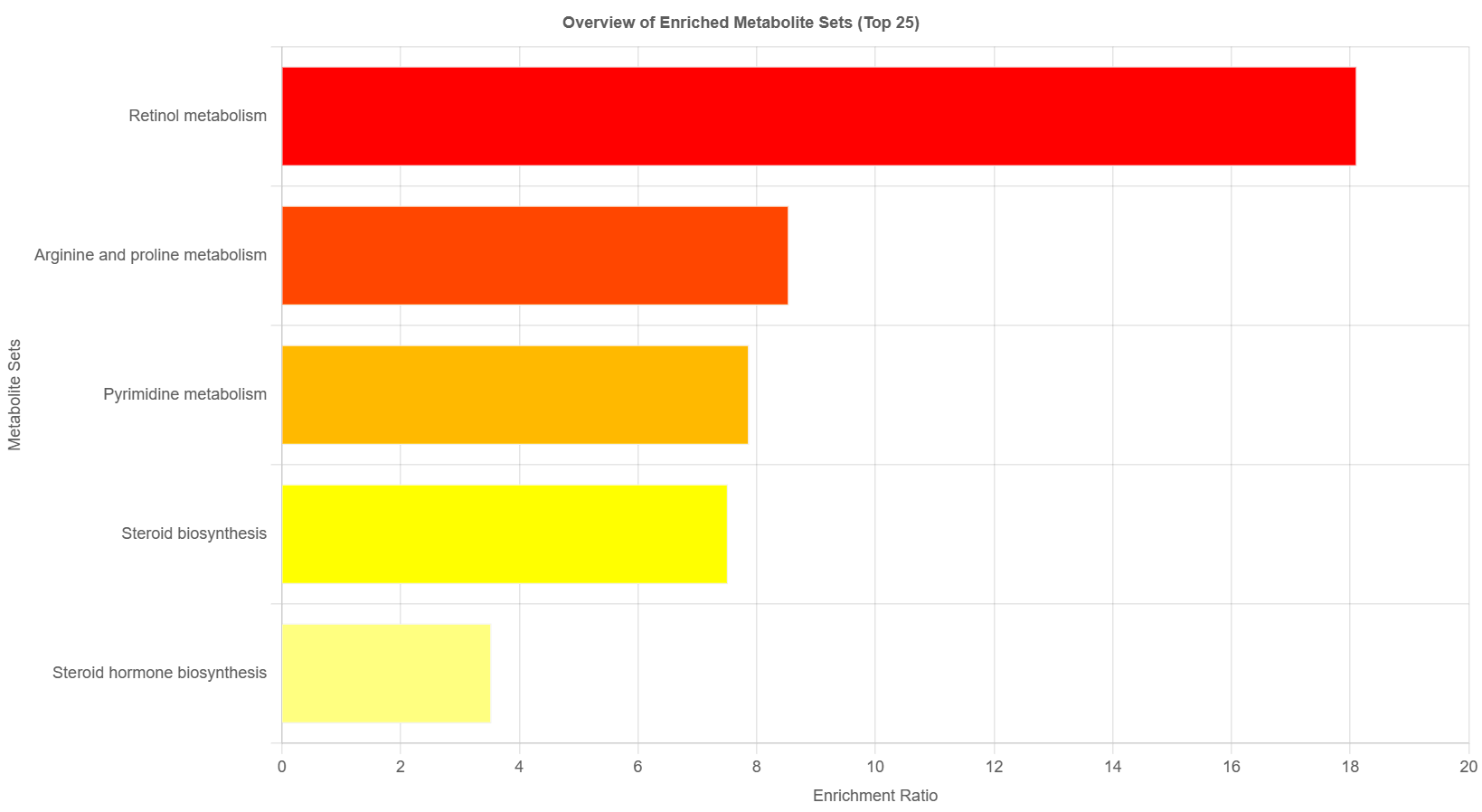
**

**D
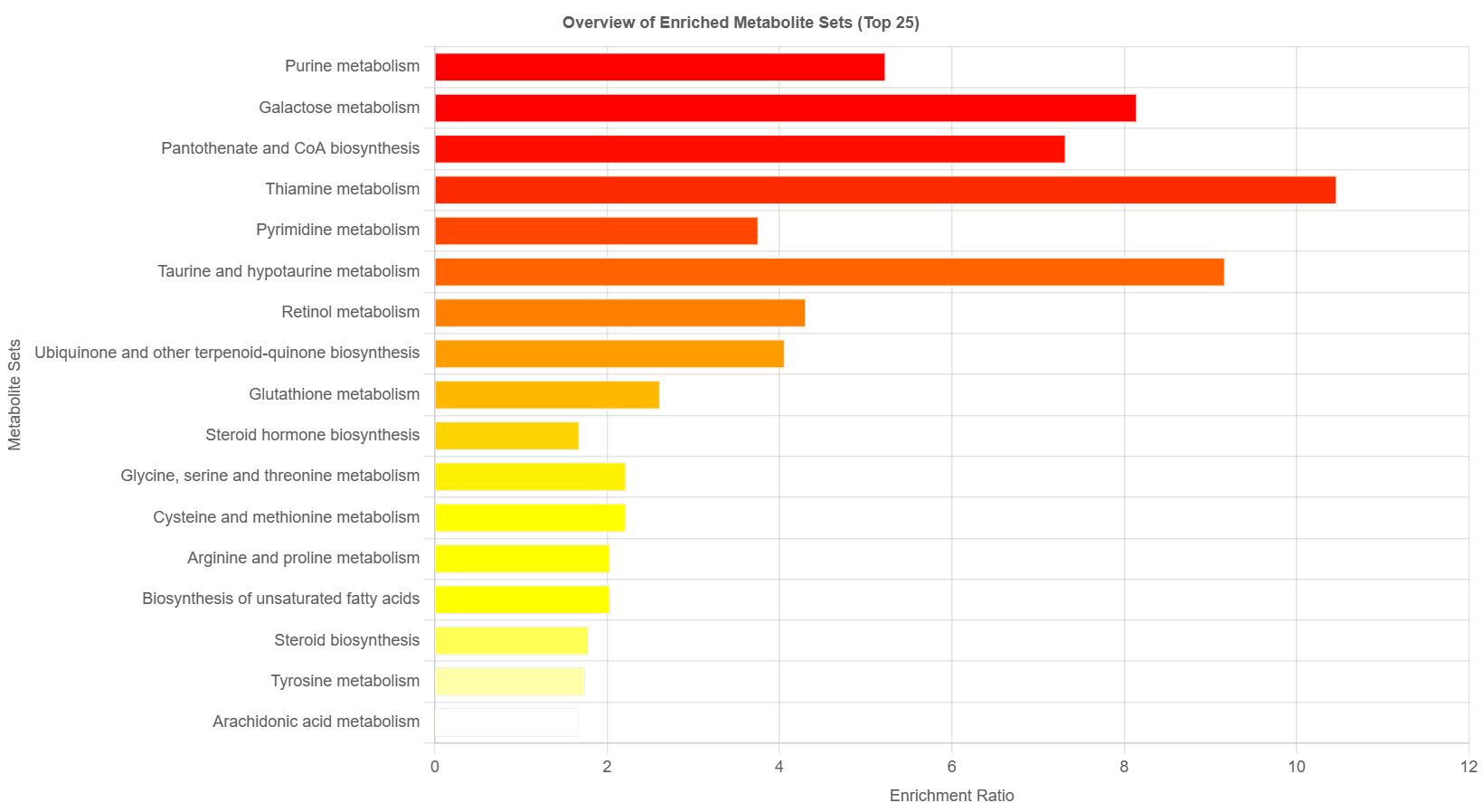
**

**E**

**
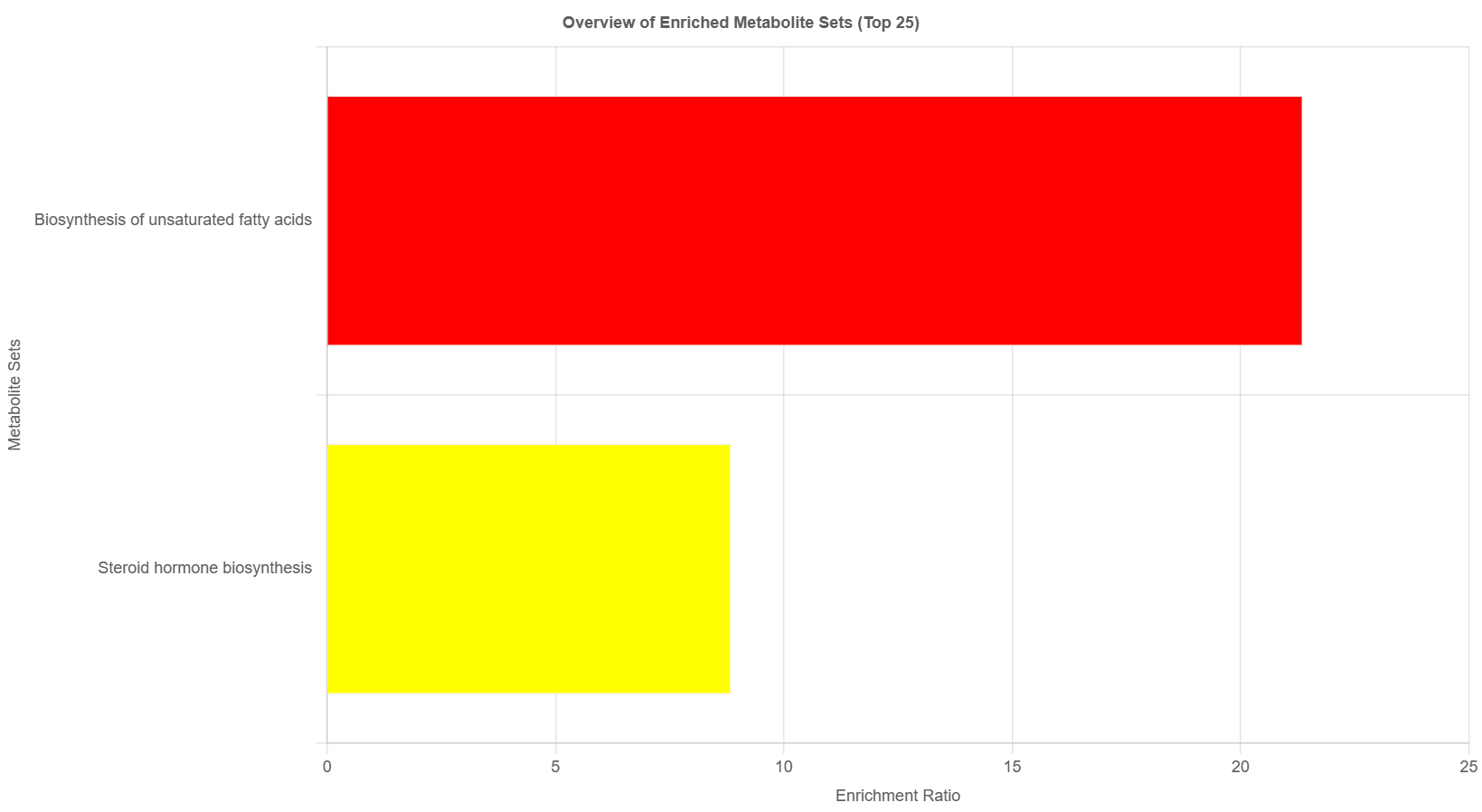
**

**S Figure 2. KEGG for the significant associated metabolites with five heavy metals.**

A) KEGG for the significant correlated metabolites with V. B). KEGG for the significant correlated metabolites with Ti. C) KEGG for the significant correlated metabolites with Cr. D)KEGG for the significant correlated metabolites with Li. E).KEGG for the significant correlated metabolites with Ni.


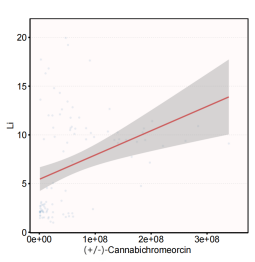

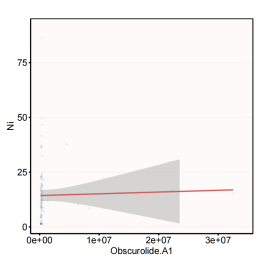

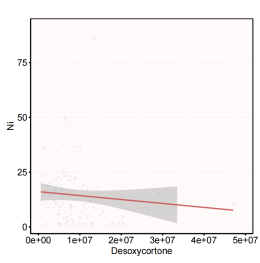

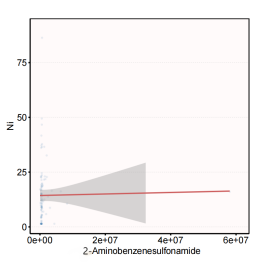

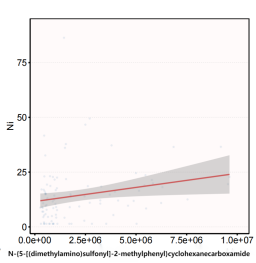

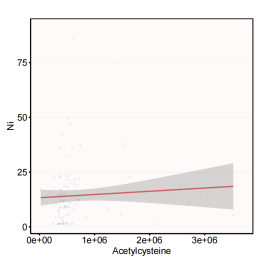

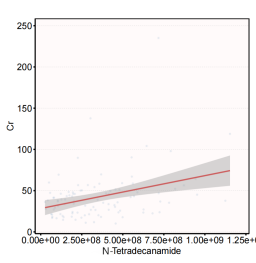

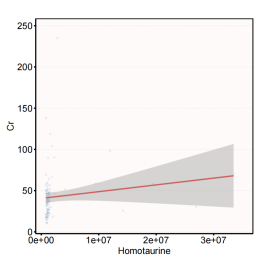

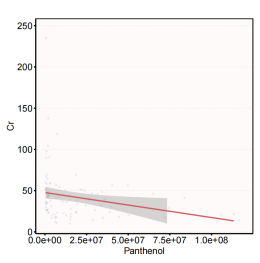

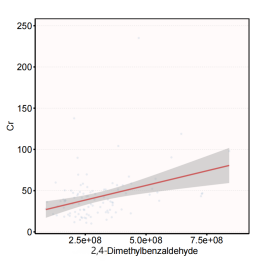

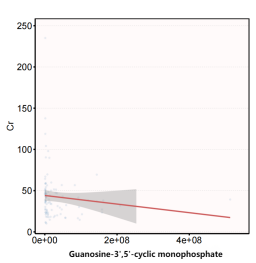

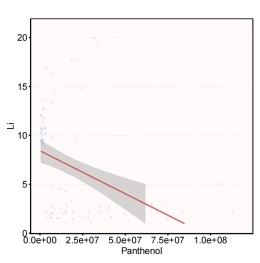

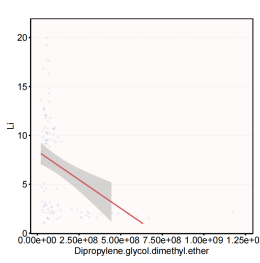

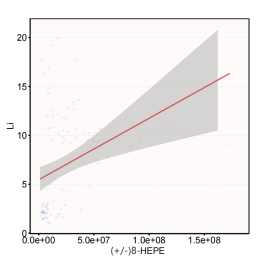

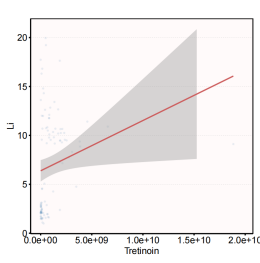

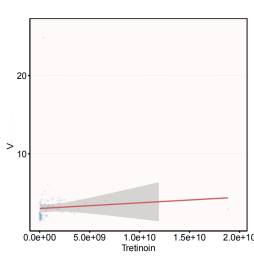

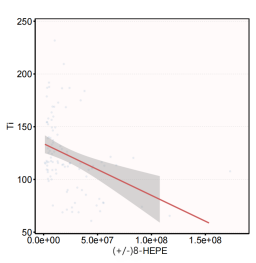

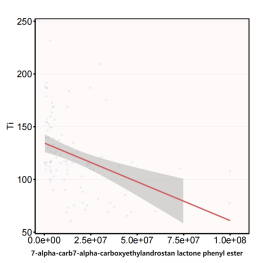

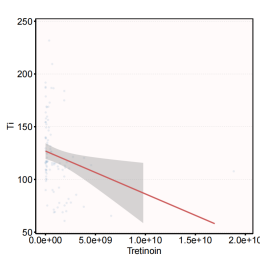

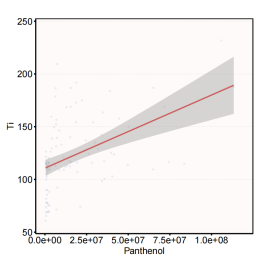

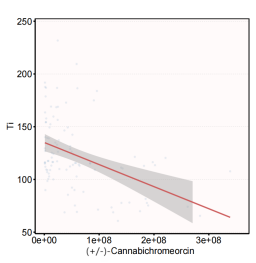

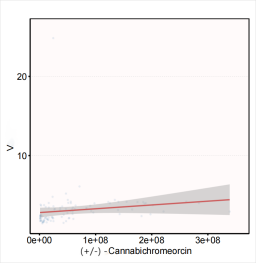

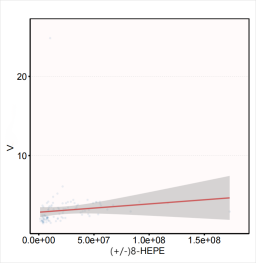

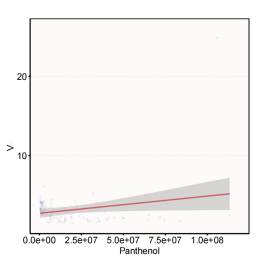

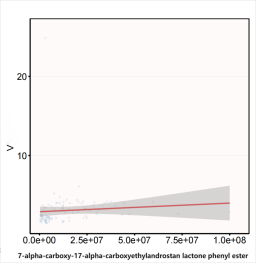


**S Figure 3. The correlations betweeen metals and metabolites .**
